# HPV Genotype Distribution in Cervical Cancer and High‐Grade Lesions in Norway, and Early Vaccine Impact

**DOI:** 10.1002/ijc.70712

**Published:** 2026-08-28

**Authors:** Terese Bekkevold, Ida Laake, Berit Feiring, Kristiane Søreng, Gro Kummeneje Presthus, Glenny Cecilie Alfsen, Irene Kraus Christiansen, Mona Hansen, Hanne Kristiansen, Eirik Olsen, Torstein Gjølgali Frengen, Emily A. Burger, Christine Genieve Monceyron Jonassen, Lill Trogstad

**Affiliations:** ^1^ Division of Infection Control Norwegian Institute of Public Health Oslo Norway; ^2^ The Norwegian HPV Reference Laboratory, Department of Microbiology and Infection Control Akershus University Hospital Lorenskog Norway; ^3^ Department of Pathology Akershus University Hospital Lorenskog Norway; ^4^ Faculty of Medicine, University of Oslo Oslo Norway; ^5^ Department of Health Management and Health Economics University of Oslo Oslo Norway; ^6^ Harvard T.H Chan School of Public Health Boston Massachusetts USA

**Keywords:** cervical cancer, high‐grade cervical lesions, human papillomavirus, human papillomavirus genotypes, human papillomavirus vaccine

## Abstract

All cases of invasive cervical cancer (ICC) and subsets of high‐grade cervical intraepithelial neoplasia grade 2 or 3 (CIN2/3) and adenocarcinoma in situ (AIS) in Norway are tested for 37 human papillomavirus (HPV) genotypes, within a mandatory national surveillance system. We assessed the proportional attribution of HPV types in cervical lesions from predominantly unvaccinated women reported during 2017–2021 (baseline population) and the HPV type distribution by vaccination status in vaccine‐eligible cases reported during 2017–2024. Among baseline CIN2/3 cases (*n* = 3087), 48% were attributable to HPV16/18, 28% to HPV31/33/45, 12% to HPV52/58 and 8% to other high‐risk types (HPV35/39/51/56/59). For AIS (*n* = 550), almost all cases were attributed to HPV16/18 (91%) and HPV45 (6%). For ICC (*n* = 1265), 75% were attributable to HPV16/18, 15% to HPV31/33/45, 3% to HPV52/58 and 4% to other high‐risk types. Among CIN2/3 cases born 1997 or later (eligible for vaccination at age 11–12 years, *n* = 154), HPV16‐attribution was higher in unvaccinated cases (49.9%, 95% CI: 33.7–66.1) than in cases vaccinated before age 15 (1.1%, 95% CI: 0–3.4). In CIN2/3 cases born 1991–1996 (eligible for catch‐up vaccination, *n* = 1541), HPV16‐attribution was 45.7% (95% CI: 41.9–49.7) in unvaccinated versus 35.5% (95% CI: 32.5–38.6) in vaccinated cases. Minor differences by vaccination status were observed for catch‐up‐eligible AIS (*n* = 445) and ICC (*n* = 135) cases. In conclusion, the baseline genotype distribution in high‐grade cervical lesions and ICC in Norway aligns with global data. Strong protection against HPV16‐induced CIN2/3 was seen in women vaccinated before age 15 years.

## What's New?

The authors present the first results from a mandatory national surveillance system on human papillomavirus (HPV) genotypes in invasive cervical cancer and high‐grade lesions in Norway. They describe the genotype distribution among predominantly unvaccinated women as a baseline for evaluating the impact of vaccination. HPV16 and HPV18 have nearly disappeared in high‐grade cervical lesions among women vaccinated with the four‐valent HPV vaccine through the Norwegian childhood immunisation programme at 11–12 years of age. Early vaccination confers strong protection against HPV16‐induced CIN2/3, and the national surveillance system is well suited for HPV genotype surveillance in the vaccine era in Norway.

## Graphical abstract

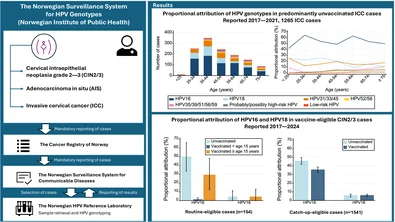

**Abbreviations**
2vHPV: 2‐valent HPV vaccine
4vHPV: 4‐valent HPV vaccine
9vHPV: 9‐valent HPV vaccine
AC: adenocarcinoma
AIS: adenocarcinoma in situ
CI: confidence interval
CIN2/3: cervical intraepithelial neoplasia grade 2 or 3
CP: crude prevalence
CRN: Cancer Registry of Norway
FFPE: formalin‐fixed paraffin‐embedded
HPV: human papillomavirus
IARC: International Agency for Research on Cancer
ICC: invasive cervical cancer
MSIS: Norwegian Surveillance System for Communicable Diseases
NIPH: Norwegian Institute of Public Health
Other ICC: other types of invasive carcinoma
PA: proportional attribution
SCC: squamous cell carcinoma
SYSVAK: Norwegian Immunisation Registry

## 1 Introduction

Invasive cervical cancer (ICC) represents a major global health burden, with approximately 600,000 new cases and 340,000 deaths annually [1]. In Norway, 269 new cases of ICC and 71 deaths were registered in 2024 [2]. ICC is caused by persistent infection with human papillomavirus (HPV) [3]. The International Agency for Research on Cancer (IARC) has classified 12 HPV genotypes as carcinogenic to humans, referred to as high‐risk types (HPV16/18/31/33/35/39/45/51/52/56/58/59), one type as probably high‐risk (HPV68) and an additional 12 types as possibly high‐risk (HPV26/30/34/53/66/67/69/70/73/82/85/97) [4]. The remaining HPV types are referred to as undetermined or low‐risk. A recent meta‐analysis confirmed the carcinogenicity of the high‐risk types and found epidemiological evidence of carcinogenicity of the probably and possibly carcinogenic types HPV68, HPV26, HPV69, HPV73 and HPV82 [5].

Early detection and removal of high‐grade cervical lesions reduce the incidence and mortality of cervical cancer [6]. Cervical screening by cytology was offered to women in Norway aged 25–69 years from 1995. From 2015, HPV‐testing has gradually replaced cytology as the primary screening modality and was expanded to include all age groups in 2023 [7].

Since 2009, HPV vaccination has been provided free of charge to girls born 1997 or later at age 11–12 years, through the Norwegian childhood immunisation programme. The uptake of the completed HPV vaccination schedule among 16‐year‐olds has gradually increased from 67% among girls born in 1997 to 93% among girls born in 2006 or later [8]. Initially, the 4‐valent vaccine (4vHPV), including antigens from HPV6/11/16/18, was offered in a 3‐dose schedule [9]. In 2017, 4vHPV was replaced by the 2‐valent vaccine (2vHPV), including antigens from HPV16/18, in a 2‐dose schedule. Boys were included in the HPV childhood immunisation programme in 2018. During 2016–2018, women born in 1991 or later (aged up to 26 years in 2016) were offered the 2vHPV in a 3‐dose schedule in a temporary catch‐up programme. The 9‐valent HPV vaccine (9vHPV), including antigens from HPV6/11/16/18/31/33/45/52/58, became available on the private market in Norway following its marketing authorisation in 2015.

As part of the national surveillance of the HPV immunisation programme, The Norwegian Institute of Public Health (NIPH), in collaboration with the Norwegian HPV Reference Laboratory, set up a system for post‐vaccination surveillance of HPV genotypes in histologically confirmed cases of ICC, cervical intraepithelial neoplasia grade 2 or 3 (CIN2/3) and adenocarcinoma in situ (AIS) in 2017 [10].

In this paper we: (1) describe the surveillance system for HPV genotypes in cervical cancer and high‐grade lesions in Norway, (2) describe the distribution of HPV genotypes in cases of CIN2/3, AIS and ICC not impacted by vaccination in Norway as a baseline for future studies evaluating the vaccine impact and (3) assess early vaccination impact on HPV genotypes in cases of CIN2/3, AIS and ICC among women eligible for vaccination.

## 2 Materials and Methods

### 2.1 HPV Genotype Surveillance

The national surveillance of HPV genotypes in high‐grade cervical lesions and cancer within the Norwegian Surveillance System for Communicable Diseases (MSIS) was made mandatory through an amendment to the MSIS‐regulations in 2014 [11]. There is mandatory reporting of all malignant neoplasms and precancerous disorders to the Cancer registry of Norway (CRN) [2]. The CRN reports all histological samples from the cervix uteri in women diagnosed with primary, invasive epithelial cancer and high‐grade dysplasia to MSIS. The ICC cases are classified as adenocarcinoma (AC), squamous cell carcinoma (SCC) or other types of invasive carcinoma (other ICC). Non‐carcinoma cancer cases are not included in the surveillance. Specimens that are retrieved at different time‐points but belong to the same disease episode are registered with the same case number [12]. A disease episode ends if there are at least two consecutive normal cytology/HPV tests or at least 2 years since the last histological sample. Any new positive histological sample after the disease episode ended is assigned a new case number. MSIS selects all ICC, a random sample (*n* = 800) of CIN2/3 and a selection of AIS cases for HPV genotyping each year. Initially, all AIS cases were included; however, due to a higher‐than‐anticipated sample volume, priority was given to samples from women with AIS aged 32 years or younger from October 2018 to capture early vaccine effectiveness. MSIS regularly transfers information on selected cases to the HPV Reference Laboratory, which requests one representative formalin‐fixed paraffin‐embedded (FFPE) block from the diagnosing pathology laboratory. For each case, the earliest cervical specimen (biopsy or cone material) with the most advanced (worst) morphology at the time of selection is requested for HPV genotyping. A case may be selected for HPV genotyping only once and any additional samples reported after the case is first selected will not be considered for re‐selection, even if later samples represent a more advanced morphology.

Four sections of 5 μm from each FFPE block are prepared for DNA extraction. Haematoxylin–eosin‐stained control sections from before and after sections cut for DNA extraction are evaluated by an experienced pathologist to confirm the histopathological diagnosis (representativeness) of the genotyped tissue. To control for contamination, clean paraffin blocks are sectioned between each sample. DNA extraction is performed using automated purification with QIAsymphony DSP DNA Mini Kit (Qiagen), after deparaffinization of tissue sections. DNA yield is measured by Nanodrop (Thermo Scientific).

HPV genotyping is performed using three sequential analyses. The first analysis is performed using a multiplex bead‐based Luminex assay which combines Modified General Primer PCR amplification with hybridisation to HPV type‐specific probes, detecting 37 HPV‐types: HPV6/11/16/18/26/30/31/33/35/39/40/42/43/45/51/52/53/54/56/58/59/61/66/67/68/69/70/73/74/81/82/83/86/87/89/90/91 (Luminex) [13]. To ensure quality and performance of the Luminex assay, the Norwegian HPV reference laboratory participates yearly in the Global HPV LabNet DNA Genotyping Proficiency Study [14]. Samples negative after Luminex are further analysed by Anyplex II HPV28/Allplex HPV28 Detection from Seegene (Allplex replaced Anyplex in April 2024) in accordance with the manufacturer's instructions, detecting 28 HPV‐types: HPV6/11/16/18/26/31/33/35/39/40/42/43/44/45/51/52/53/54/56/58/59/61/66/68/69/70/73/82 (Anyplex/Allplex). Samples still negative for HPV are analysed by an in‐house type‐specific real‐time PCR targeting the E6E7 genes of 14 HPV types in the following order: HPV16, HPV18, HPV45, HPV31, HPV33, HPV52, HPV58, HPV35, HPV39, HPV51, HPV56, HPV59, HPV68 and HPV66 (E6/E7). When a positive result is obtained, no further analysis is undertaken. Each type is assessed in singleplex reactions with 5 μL DNA input, using SYBR Green as a fluorescent dye. Primer sequences were obtained from published sources [15, 16, 17, 18] and amplification conditions were optimised in‐house (Tables S1 and S2). Primers for HPV68 were designed in‐house.

Information on HPV positive cases, including results from genotyping, is stored in MSIS [19]. Details on the data retrieved from MSIS are presented in Table S3.

### 2.2 Vaccination Status

For this study, we linked the HPV genotype information in MSIS to the Norwegian Immunisation Registry (SYSVAK) using the personal ID allocated to all residents of Norway [20]. Cases were categorised as ‘non‐vaccine‐eligible’ (born in 1990 or earlier), ‘catch‐up‐eligible’ (born 1991–1996) or ‘routine‐eligible’ (born in 1997 or later). A case was considered ‘vaccinated’ if at least one dose of HPV vaccine was administered more than 365 days before they were first diagnosed with CIN2/3, AIS or ICC and ‘unvaccinated’ otherwise. As the data did only include birth years, we could not calculate the exact age at vaccination. Routine‐eligible vaccinated cases were considered ‘vaccinated before age 15 years’ if the first dose of HPV vaccine was administered prior to the year they turned 15 years, which for most would be before their sexual debut [21]. Those who received the first vaccine dose the year they turned 15 years or later were considered ‘vaccinated at age 15 years or later’.

### 2.3 Baseline Population

To describe the genotype distribution in unvaccinated cases of CIN2/3, AIS and ICC, we included cases reported to MSIS during 2017–2021, who tested positive for at least one HPV genotype and with at least one representative control section (baseline population). We expect few cases to be impacted by vaccination prior to 2022 for two reasons: (1) most cases vaccinated in the catch‐up programme, implemented less than 5 years before the study period ended, likely contracted the causal infection prior to vaccination, and (2) cohorts eligible for vaccination at age 11–12 years did not enter the cervical screening programme until 2022.

### 2.4 Vaccine Impact Population

For the assessment of vaccine impact, we included the vaccine‐eligible cases (born 1991 or later) reported to MSIS during 2017–2024, who tested positive for at least one HPV genotype and with at least one representative control section (vaccine impact population). Genotyping of cases reported during 2024 was not completed at the time of data retrieval, but we included genotyped cases from 2024 to have as many vaccinated cases as possible in the dataset. We assumed that any difference between genotyped and not genotyped cases reported during 2024 would be random, as cases are genotyped in the order that their sample is reported and retrieved.

### 2.5 Statistical Analysis

In the baseline population, we calculated the genotype distribution separately for cases with CIN2/3, AIS and ICC, and for the ICC sub‐morphologies SCC, AC and other ICC. For each of the 37 individual genotypes included in the Luminex assay, we calculated the crude prevalence (CP) with Wilson score 95% confidence intervals (CIs). Genotype‐specific positive results in single or multiple infections contribute equally to the CP, and the total CP will consequently exceed 100%. Since each lesion is presumably caused by one genotype only, the CP likely overestimates the causal contribution of some genotypes [22]. Thus, we also calculated the proportional attribution (PA). The total PA for each morphology adds up to 100%, since each genotype‐specific positive result in multiple infections was weighted according to its proportion in single infections within the same morphology [23, 24, 25]. We performed nonparametric bootstrap estimation of the CIs for the PAs. For each morphology, we generated 1000 bootstrap samples, drawing cases with replacement from the original data set. For each bootstrap sample, we calculated the PA, using weights derived from the bootstrap sample. The 95% CI was defined as the interval between the 2.5th percentile and the 97.5th percentile of the sample PAs. Additionally, we calculated the genotype distribution using two alternative methods: (1) the single infection approach (genotype‐specific prevalence among cases with single infection only), and (2) the hierarchical approach (in cases with multiple infections, only the most prevalent type for the morphology contributes to the prevalence) [24, 25].

Furthermore, we calculated the CP and PA in the baseline population for the following groups of genotypes by morphology: (1) HPV16/18, types with direct protection from 2vHPV, 4vHPV and 9vHPV; (2) HPV31/33/45, types with direct protection from 9vHPV and evidence of some cross‐protection from 2vHPV [26] and 4vHPV [27] (3) HPV52/58, types covered by 9vHPV and not covered by 2vHPV or 4vHPV; (4) HPV35/39/51/56/59, high‐risk types not covered by any vaccine; (5) HPV16/18/31/33/35/39/45/51/52/56/58/59, all high‐risk types; (6) HPV26/30/53/66/67/68/69/70/73/82, probably and possibly high‐risk types; (7) HPV6/11/40/42/43/54/61/74/81/83/86/87/89/90/91, low‐risk types. The CP of a group of genotypes equals the CP of cases positive for one or more types in the group, while the PAs of the individual genotypes were summed for the total group PAs.

For CIN2/3 and ICC in the baseline population, we calculated the PA of HPV16, HPV18, HPV31/33/45, HPV52/58, HPV35/39/51/56/59, probably/possibly high‐risk types and low‐risk types by age categories. For CIN2/3 we used the age categories < 25, 25–34, 35–44, 45–54 and ≥ 55 years. Due to a higher incidence of ICC in older age, we used the following age categories for ICC: < 25, 25–34, 35–44, 45–54, 55–64, 65–74 and ≥ 75 years. The PAs of HPV genotypes by age were not calculated for AIS, since these cases were selected for HPV genotyping according to their birth year. As HPV vaccination could impact the genotype distribution, we also calculated the PAs by age groups after excluding vaccinated cases.

For the morphologies CIN2/3, AIS and ICC in the baseline population, we compared the CP of individual genotypes in cases where the histopathological diagnosis was confirmed in at least one control section (confirmed cases) to the CP in cases where the histopathological diagnosis was not confirmed in either of the two control sections (unconfirmed cases). We compared CPs since the weights used to calculate the PA are based on prevalence in confirmed cases only. Groups were compared using Fisher's exact test and corresponding mid‐*p*‐values. A mid‐*p*‐value less than 0.05 was considered statistically significant. The test was only performed for high‐risk types and types for which the total number of genotype‐specific positive cases across the two groups was higher than 10. As a sensitivity analysis, we calculated the PA of individual HPV genotypes without excluding unconfirmed cases.

Among the catch‐up‐eligible cases in the vaccine impact population, we calculated the PA of genotypes for vaccinated and unvaccinated cases of CIN2/3, AIS and ICC, using weights from the baseline population. Among the routine‐eligible CIN2/3 cases in the vaccine impact population, we calculated the PA of genotypes separately for unvaccinated cases, cases vaccinated before age 15 years, and cases vaccinated at age 15 years or later. The 95% CIs for the PAs were calculated in the same manner as for the baseline population, except that weights from the baseline population were used to calculate all of the bootstrap PAs instead of weights derived from each bootstrap sample. Genotypes by vaccination status were not assessed for AIS and ICC due to the low number of cases.

## 3 Results

### 3.1 Baseline Population

During 2017–2021, 5787 cases were selected for HPV genotyping (Figure 1A). Of these, 5513 cases were genotyped, and 97.0% of CIN2/3, 95.8% of AIS, and 95.2% of ICC cases tested positive for at least one HPV genotype.

**FIGURE 1:**
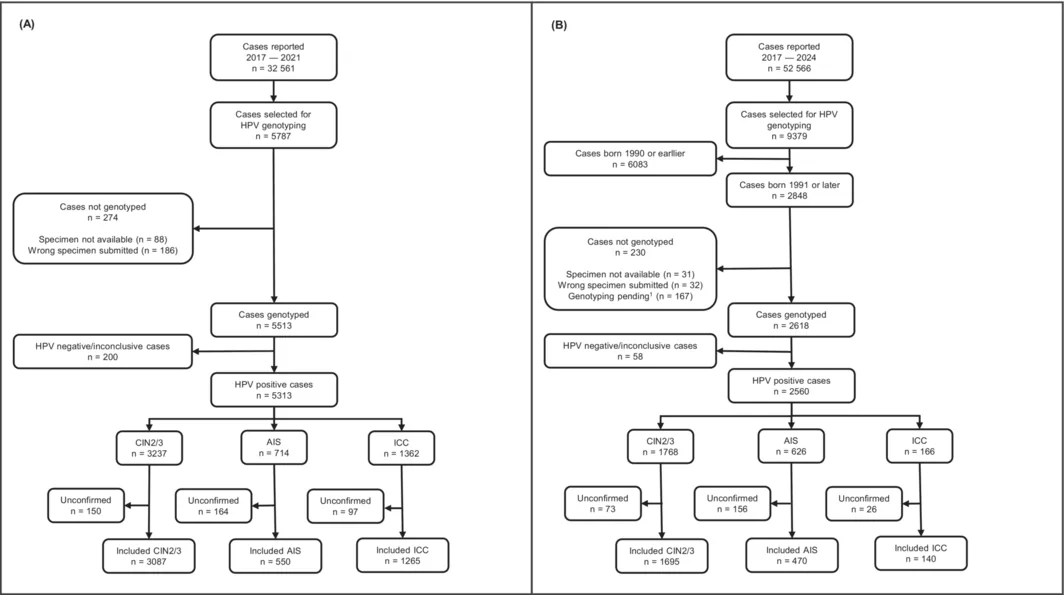
Flowchart of cases included in the two study populations. (A) Baseline population (B) Vaccine impact population. AIS, adenocarcinoma in situ; CIN2/3, cervical intraepithelial neoplasia grade 2 or 3; ICC, invasive cervical cancer, Unconfirmed: Cases where the histopathological diagnosis could not be confirmed in either of the haematoxylin–eosin‐stained control sections. ^1^One case reported during 2022, 1 case reported during 2023 and 165 cases reported during 2024.

Among ICC cases, the proportion of HPV positives was lower among other ICC (83.5%) and AC (89.0%) than for SCC (97.6%). After exclusion of unconfirmed cases, 3087 CIN2/3, 550 AIS and 1265 ICC cases were included in the baseline genotype distribution assessment.

Median age of diagnosis was 32 years for CIN2/3, 28 years for AIS and 45 years for ICC. The majority of CIN2/3 and ICC cases were non‐vaccine‐eligible (71.8% and 93.3%, respectively), while 51.8% of AIS cases were catch‐up‐eligible (Table 1). For all morphologies, < 1% of cases were routine‐eligible. The majority of cases were unvaccinated (84.7%, 68.2% and 96.5% of CIN2/3, AIS and ICC cases, respectively). No more than 0.5% of cases of any morphology were vaccinated before age 15 years. Among those aged < 25 years, nearly half of CIN2/3 (46.9%), 30.2% of AIS and 50.0% of ICC cases (totaling 12 cases only) had been vaccinated, while in the 25–34‐year age group, the percentage of vaccinated was 22.9% for CIN2/3, 38.5% for AIS and 15.3% for ICC (Table S4). In the age groups ≥ 35 years, only 0%–1.5% were vaccinated.

**TABLE 1:** Characteristics of specimens included in the study, separately for the baseline population (cases reported during 2017–2021 and including all birth cohorts) and the vaccine impact population (cases reported during 2017–2024 and born 1991 or later).

	Baseline population			Vaccine impact population		
	Reported 2017–2021			Reported 2017–2024		
				Born 1991 or later		
	CIN2/3	AIS	ICC	CIN2/3	AIS	ICC
	*N* = 3087	*N* = 550	*N* = 1265	*N* = 1695	*N* = 470	*N* = 140
	*n* (%)	*n* (%)	*n* (%)	*n* (%)	*n* (%)	*n* (%)
Age at diagnosis						
< 25 years	162 (5.2)	43 (7.8)	12 (0.9)	220 (13.0)	46 (9.8)	14 (10.0)
25–34 years	1666 (54.0)	421 (76.5)	248 (19.6)	1475 (87.0)	424 (90.2)	126 (90.0)
35–44 years	724 (23.5)	62 (11.3)	346 (27.4)	—	—	—
45–54 years	336 (10.9)	24 (4.4)	241 (19.1)	—	—	—
55–64 years	127 (4.1)	NA^d^	188 (14.9)	—	—	—
65–74 years	67 (2.2)	NA^d^	145 (11.5)	—	—	—
75 years or older	5 (0.2)	NA^d^	85 (6.7)	—	—	—
Birthyear						
1990 or earlier (non‐vaccine‐eligible)	2216 (71.8)	265 (48.2)	1180 (93.3)	—	—	—
1991–1996 (catch‐up‐eligible)	842 (27.3)	285 (51.8)	85 (6.7)	1541 (90.9)	445 (94.7)	135 (96.4)
1997 or later (routine‐eligible)	29 (0.9)	NA^e^	NA^e^	154 (9.1)	25 (5.3)	5 (3.6)
Vaccination status						
Unvaccinated^a^	2614 (84.7)	375 (68.2)	1221 (96.5)	703 (41.5)	170 (36.2)	62 (44.3)
Vaccinated before age 15 years^b^	14 (0.5)	NA^f^	0 (0.0)	98 (5.8)	NA^f^	NA^f^
Vaccinated at age 15 years or later^c^	459 (14.9)	175 (31.8)	44 (3.5)	894 (52.7)	300 (63.8)	78 (55.7)

Abbreviations: AIS, adenocarcinoma in situ; CIN2/3, cervical intraepithelial neoplasia grade 2 or 3; ICC, invasive cervical cancer.

^a^ Unvaccinated: not registered with any HPV vaccine dose or received the first dose 365 days or less before they were first diagnosed with CIN2/3, AIS or ICC.

^b^ Vaccinated before age 15 years: received at least one dose of HPV vaccine 365 days or more before they were first diagnosed with CIN2/3, AIS or ICC and prior to the year they turned 15 years old.

^c^ Vaccinated at age 15 years or later: received at least one dose of HPV vaccine 365 days or more before they were first diagnosed with CIN2/3, AIS or ICC, with the first dose administered the year they turned 15 years or later.

^d^ Since there were fewer than five cases in the age groups older than 54 years, they were anonymized by reporting them together with AIS cases 45–54 years.

^e^ Since there were fewer than five routine‐eligible cases, they were anonymized by reporting them together with catch‐up eligible cases.

^f^ Since fewer than five cases were vaccinated before age 15 years, they were anonymized by reporting them together with cases vaccinated at age 15 years or later.

### 3.2 Baseline Genotype Distribution

In 81.2% (95% CI: 79.8–82.5) of CIN2/3, 78.4% (95% CI: 74.7–81.6) of AIS and 96.4% (95% CI: 95.3–97.3) of ICC cases, only one HPV genotype was detected (Table 2). For CIN2/3, the eight types with the highest PA, in descending order, were HPV16, HPV31, HPV33, HPV52, HPV18, HPV58, HPV45 and HPV51. The PAs of the genotype groups HPV16/18, HPV31/33/45, HPV52/58 and other high‐risk types (HPV35/39/51/56/59) were 47.8% (95% CI: 46.0–49.5), 27.8% (95% CI: 26.3–29.6), 11.5% (95% CI: 10.4–12.6) and 8.1% (95% CI: 7.2–9.1), respectively, totaling 95.2% (95% CI: 94.4–96.0) for all high‐risk types. For AIS, 96.9% (95% CI: 95.4–98.3) of cases were attributed to the three most prevalent types, HPV18, HPV16 and HPV45, and the combined PA of HPV16/18 was 90.7% (95% CI: 88.1–93.2). For ICC cases, the eight types with the highest PA were (in descending order), HPV16, HPV18, HPV45, HPV33, HPV31, HPV52, HPV73 and HPV39. The PAs of the genotype groups HPV16/18, HPV31/33/45, HPV52/58 and HPV35/39/51/56/59 were 74.6% (95% CI: 72.1–77.1), 15.3% (95% CI: 13.4–17.2), 2.5% (95% CI: 1.7–3.5) and 4.0% (95% CI: 2.9–5.2), respectively, totaling 96.4% (95% CI: 95.4–97.5) for all high‐risk types. The eight most prevalent types in SCC mirrored those for ICC, except that HPV33 was the third most prevalent in SCC, followed by HPV45 as the fourth (Table S5). AC and other ICC were predominantly attributed to HPV18 and HPV16.

**TABLE 2:** Crude prevalence and proportional attribution of Human papillomavirus (HPV) genotypes by morphology, in the baseline population reported during 2017–2021.

	Crude prevalence						Proportional attribution					
	CIN2/3		AIS		ICC		CIN2/3		AIS		ICC	
	*N* = 3087		*N* = 550		*N* = 1265		*N* = 3087		*N* = 550		*N* = 1265	
	*n*	% (95% CI)	*n*	% (95% CI)	*n*	% (95% CI)	*n* ^a^	% (95% CI^b^)	*n* ^a^	% (95% CI^b^)	*n* ^a^	% (95% CI^b^)
Number of HPV types												
1	2506	81.2 (79.8–82.5)	431	78.4 (74.7–81.6)	1220	96.4 (95.3–97.3)		—		—		—
2	467	15.1 (13.9–16.4)	109	19.8 (16.7–23.4)	41	3.2 (2.4–4.4)		—		—		—
≥ 3	114	3.7 (3.1–4.4)	10	1.8 (1.0–3.3)	4	0.3 (0.1–0.8)		—		—		—
High‐risk HPV types												
16	1303	42.2 (40.5–44.0)	224	40.7 (36.7–44.9)	704	55.7 (52.9–58.4)	1268.7	41.1 (39.5–42.8)	197.0	35.8 (31.7–40.1)	702.0	55.5 (52.9–58.3)
18	270	8.7 (7.8–9.8)	321	58.4 (54.2–62.4)	248	19.6 (17.5–21.9)	206.8	6.7 (5.8–7.6)	301.9	54.9 (50.7–59.1)	241.7	19.1 (17.0–21.3)
31	558	18.1 (16.8–19.5)	32	5.8 (4.2–8.1)	53	4.2 (3.2–5.4)	488.1	15.8 (14.5–17.2)	4.7	0.9 (0.2–1.8)	48.5	3.8 (2.9–5.0)
33	304	9.8 (8.8–10.9)	7	1.3 (0.6–2.6)	67	5.3 (4.2–6.7)	264.3	8.6 (7.6–9.6)	0	0 (NA)	67.0	5.3 (4.1–6.6)
35	95	3.1 (2.5–3.7)	3	0.5 (0.2–1.6)	10	0.8 (0.4–1.4)	61.7	2.0 (1.5–2.5)	0	0 (NA)	8.6	0.7 (0.2–1.2)
39	79	2.6 (2.1–3.2)	6	1.1 (0.5–2.4)	16	1.3 (0.8–2.0)	53.2	1.7 (1.2–2.2)	1.0	0.2 (0–0.6)	15.0	1.2 (0.6–1.8)
45	132	4.3 (3.6–5.0)	37	6.7 (4.9–9.1)	81	6.4 (5.2–7.9)	106.6	3.5 (2.8–4.2)	34.2	6.2 (4.2–8.4)	78.3	6.2 (4.9–7.5)
51	123	4.0 (3.3–4.7)	5	0.9 (0.4–2.1)	3	0.2 (0.08–0.7)	78.1	2.5 (2.0–3.1)	0	0 (NA)	1.0	0.08 (0–0.2)
52	299	9.7 (8.7–10.8)	11	2.0 (1.1–3.5)	25	2.0 (1.3–2.9)	234.5	7.6 (6.7–8.6)	0	0 (NA)	24.7	2.0 (1.2–2.8)
56	57	1.8 (1.4–2.4)	4	0.7 (0.3–1.9)	15	1.2 (0.7–1.9)	34.6	1.1 (0.8–1.6)	1.0	0.2 (0–0.6)	14.9	1.2 (0.6–1.8)
58	150	4.9 (4.2–5.7)	3	0.5 (0.2–1.6)	10	0.8 (0.4–1.4)	120.0	3.9 (3.2–4.6)	0	0 (NA)	7.3	0.6 (0.2–1.1)
59	41	1.3 (1.0–1.8)	5	0.9 (0.4–2.1)	13	1.0 (0.6–1.8)	23.2	0.8 (0.5–1.1)	2.0	0.4 (0–0.9)	11.1	0.9 (0.4–1.5)
Probably/possibly high‐risk types												
26	6	0.2 (0.09–0.4)	0	0 (0–0.7)	0	0 (0–0.3)	2.2	0.07 (0–0.2)	0	0 (NA)	0	0 (NA)
30	16	0.5 (0.3–0.8)	0	0 (0–0.7)	3	0.2 (0.08–0.7)	4.8	0.2 (0.04–0.3)	0	0 (NA)	2.1	0.2 (0–0.4)
53	38	1.2 (0.9–1.7)	1	0.2 (0.03–1.0)	1	0.08 (0.01–0.4)	18.4	0.6 (0.3–0.9)	0	0 (NA)	0	0 (NA)
66	39	1.3 (0.9–1.7)	2	0.4 (0.10–1.3)	1	0.08 (0.01–0.4)	21.2	0.7 (0.4–1.0)	1.0	0.2 (0–0.6)	1.0	0.08 (0–0.2)
67	29	0.9 (0.7–1.3)	2	0.4 (0.10–1.3)	2	0.2 (0.04–0.6)	11.4	0.4 (0.2–0.6)	0	0 (NA)	2.0	0.2 (0–0.4)
68	33	1.1 (0.8–1.5)	2	0.4 (0.10–1.3)	10	0.8 (0.4–1.4)	14.4	0.5 (0.2–0.8)	1.0	0.2 (0–0.6)	9.0	0.7 (0.2–1.2)
69	4	0.1 (0.05–0.3)	0	0 (0–0.7)	0	0 (0–0.3)	1.0	0.03 (0–0.10)	0	0 (NA)	0	0 (NA)
70	27	0.9 (0.6–1.3)	5	0.9 (0.4–2.1)	7	0.6 (0.3–1.1)	13.6	0.4 (0.2–0.7)	3.0	0.6 (0–1.3)	6.0	0.5 (0.2–0.9)
73	11	0.4 (0.2–0.6)	0	0 (0–0.7)	20	1.6 (1.0–2.4)	5.2	0.2 (0.03–0.3)	0	0 (NA)	18.6	1.5 (0.8–2.2)
82	56	1.8 (1.4–2.3)	0	0 (0–0.7)	11	0.9 (0.5–1.6)	29.1	0.9 (0.6–1.3)	0	0 (NA)	1.2	0.09 (0–0.3)
Low‐risk types												
6	28	0.9 (0.6–1.3)	3	0.5 (0.2–1.6)	4	0.3 (0.1–0.8)	10.3	0.3 (0.1–0.6)	1.0	0.2 (0–0.6)	1.0	0.08 (0–0.2)
11	11	0.4 (0.2–0.6)	1	0.2 (0.03–1.0)	0	0 (0–0.3)	3.1	0.1 (0–0.2)	0	0 (NA)	0	0 (NA)
40	4	0.1 (0.05–0.3)	0	0 (0–0.7)	2	0.2 (0.04–0.6)	0	0 (NA)	0	0 (NA)	0	0 (NA)
42	16	0.5 (0.3–0.8)	1	0.2 (0.03–1.0)	4	0.3 (0.1–0.8)	4.9	0.2 (0.04–0.3)	0	0 (NA)	3.1	0.2 (0–0.6)
43	9	0.3 (0.2–0.6)	0	0 (0–0.7)	1	0.08 (0.01–0.4)	0	0 (NA)	0	0 (NA)	0	0 (NA)
54	4	0.1 (0.05–0.3)	0	0 (0–0.7)	0	0 (0–0.3)	0	0 (NA)	0	0 (NA)	0	0 (NA)
61	3	0.10 (0.03–0.3)	1	0.2 (0.03–1.0)	0	0 (0–0.3)	1.0	0.03 (0–0.10)	1.0	0.2 (0–0.5)	0	0 (NA)
74	10	0.3 (0.2–0.6)	0	0 (0–0.7)	0	0 (0–0.3)	0	0 (NA)	0	0 (NA)	0	0 (NA)
81	6	0.2 (0.09–0.4)	2	0.4 (0.10–1.3)	0	0 (0–0.3)	3.3	0.1 (0–0.2)	1.0	0.2 (0–0.6)	0	0 (NA)
83	10	0.3 (0.2–0.6)	0	0 (0–0.7)	0	0 (0–0.3)	2.2	0.07 (0–0.2)	0	0 (NA)	0	0 (NA)
86	1	0.03 (0.006–0.2)	1	0.2 (0.03–1.0)	0	0 (0–0.3)	0	0 (NA)	0	0 (NA)	0	0 (NA)
87	6	0.2 (0.09–0.4)	0	0 (0–0.7)	1	0.08 (0.01–0.4)	0	0 (NA)	0	0 (NA)	0	0 (NA)
89	3	0.10 (0.03–0.3)	0	0 (0–0.7)	0	0 (0–0.3)	0	0 (NA)	0	0 (NA)	0	0 (NA)
90	5	0.2 (0.07–0.4)	0	0 (0–0.7)	0	0 (0–0.3)	0	0 (NA)	0	0 (NA)	0	0 (NA)
91	19	0.6 (0.4–1.0)	0	0 (0–0.7)	2	0.2 (0.04–0.6)	1.2	0.04 (0–0.1)	0	0 (NA)	1.0	0.08 (0–0.2)
Groups of high‐risk HPV types												
16/18	1536	49.8 (48.0–51.5)	500	90.9 (88.2–93.0)	945	74.7 (72.2–77.0)	1475.5	47.8 (46.0–49.5)	499.0	90.7 (88.1–93.2)	943.6	74.6 (72.1–77.1)
31/33/45	973	31.5 (29.9–33.2)	74	13.5 (10.9–16.6)	201	15.9 (14.0–18.0)	859.0	27.8 (26.3–29.6)	38.9	7.1 (4.9–9.4)	193.8	15.3 (13.4–17.2)
52/58	445	14.4 (13.2–15.7)	14	2.5 (1.5–4.2)	35	2.8 (2.0–3.8)	354.5	11.5 (10.4–12.6)	0	0 (NA)	32.0	2.5 (1.7–3.5)
35/39/51/56/59	378	12.2 (11.1–13.4)	22	4 (2.7–6.0)	57	4.5 (3.5–5.8)	250.8	8.1 (7.2–9.1)	4.1	0.7 (0–1.5)	50.6	4.0 (2.9–5.2)
HPV types grouped by risk‐category												
High‐risk	2952	95.6 (94.8–96.3)	542	98.5 (97.2–99.3)	1222	96.6 (95.5–97.5)	2939.8	95.2 (94.4–96.0)	542.0	98.5 (97.4–99.5)	1220.0	96.4 (95.4–97.5)
Probably/possibly high‐risk	248	8 (7.1–9.0)	12	2.2 (1.3–3.8)	55	4.3 (3.4–5.6)	121.3	3.9 (3.2–4.7)	5.0	0.9 (0.2–1.8)	39.9	3.2 (2.2–4.2)
Low‐risk	127	4.1 (3.5–4.9)	9	1.6 (0.9–3.1)	14	1.1 (0.7–1.8)	25.9	0.8 (0.5–1.2)	3.0	0.6 (0–1.3)	5.1	0.4 (0.08–0.8)

Abbreviations: AIS, adenocarcinoma in situ; CI, confidence Interval; CIN2/3, cervical intraepithelial neoplasia grade 2 or 3; ICC, invasive cervical cancer.

^a^ Numbers are not whole due to weighting.

^b^ 95% CI not calculated when weighted number of cases is 0.

Among the 5028 HPV genotyped and confirmed cases, 457 (9.1%) tested negative by the initial Luminex (data not shown). Of these, 331 cases (72.4%) tested positive after subsequent testing with Anyplex/Allplex or E6/E7. Of the HPV positive CIN2/3 cases, 2834 (91.8%) were positive by Luminex, while 224 (7.3%) tested negative by Luminex and positive by Anyplex/Allplex and 29 (0.9%) tested negative by Luminex and Anyplex/Allplex but positive by E6/E7. Of the HPV positive AIS cases, 529 (96.2%) were positive by Luminex, while 18 (3.3%) tested negative by Luminex and positive by Anyplex/Allplex and 3 (0.5%) tested negative by Luminex and Anyplex/Allplex but positive by E6/E7. Of the HPV positive ICC cases, 1208 (95.5%) were positive by Luminex, 47 (3.7%) tested negative by Luminex and positive by Anyplex/Allplex and 10 (0.8%) tested negative by Luminex and Anyplex/Allplex but positive by E6/E7. For all morphologies, the CPs of HPV genotypes with the full three‐sequential testing regime remained similar to the CPs after testing by Luminex only (Table S6).

The CP for individual genotypes was consistently equal to or higher compared to the PAs, but for all morphologies, the difference was less than 5 percentage points (Table 2). For all morphologies, prevalences calculated using the single infections approach and hierarchical approach were similar to the PAs (Table S7).

The number of CIN2/3 cases in the age groups < 25, 25–34, 35–44, 45–54 and 55 years or older, were 162, 1666, 724, 336 and 199, respectively (Figure 2A and Table S8). HPV16 accounted for the highest number of cases in all age groups. The number of cases attributable to HPV16 and HPV18 was highest in the age group 25–34 years and thereafter decreased by age. The same pattern was observed for HPV31/33/45, HPV52/58, HPV35/39/51/56/59 and probably/possibly high‐risk types. The highest PA of HPV16 was observed in CIN2/3 cases aged 25–34 years (45.1%, 95% CI: 42.9–47.5) and estimates varied between 29.7% and 38.7% in the other age groups (Figure 2B and Table S8). Across age groups, the PA varied between 3.7% and 7.9% for HPV18, 25.4%–29.2% for HPV31/33/45, 10.4%–14.2% for HPV52/58 and 6.7%–14.6% for HPV35/39/51/56/59, with overlapping 95% CIs by genotype groups. Excluding vaccinated CIN2/3 cases had little impact on the PAs of genotypes in all age groups.

**FIGURE 2:**
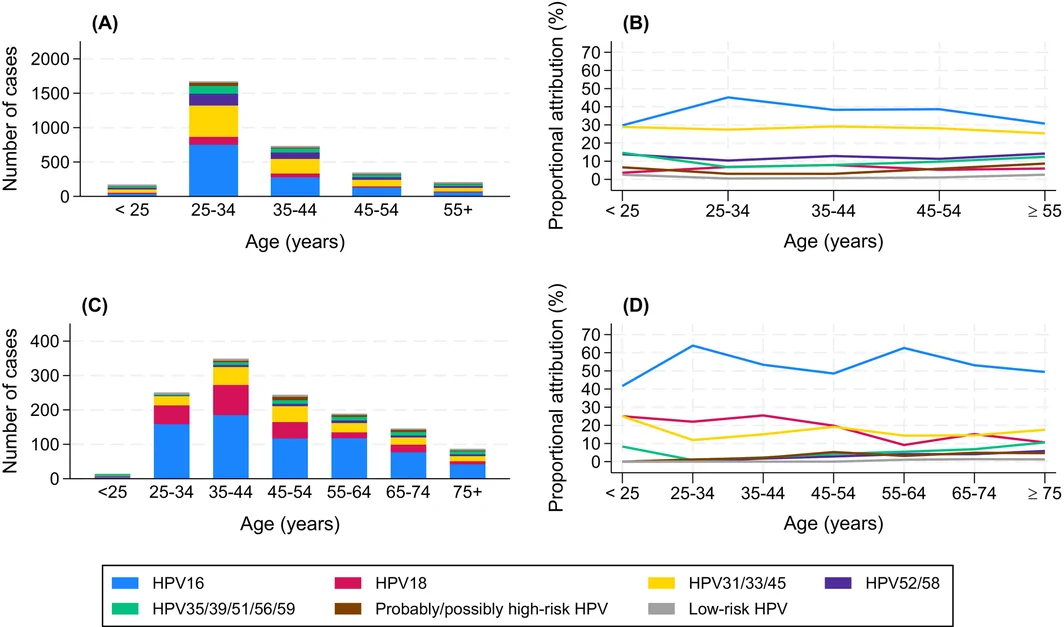
Open in figure viewer Genotype distribution by age in cases reported during 2017–2021 (baseline population). (A) Number of cervical intraepithelial neoplasia (CIN) grade 2 or 3 cases per HPV genotype category by age groups. (B) Proportional attributions of HPV genotype categories to CIN2/3 cases by age groups. (C) Number of cases of invasive cervical cancer (ICC) per HPV genotype category by age groups. (D) Proportional attributions of HPV genotype categories to ICC cases by age groups. Probably/possibly high‐risk HPV: HPV26/30/53/66/67/68/69/70/73/82. Low‐risk HPV: HPV6/11/40/42/43/54/61/74/81/83/86/87/89/90/91.

The number of ICC cases in the age groups < 25, 25–34, 35–44, 45–54, 55–64, 65–74 and 75 years or older were 12, 248, 346, 241, 188, 145 and 85, respectively (Figure 2C and Table S9). HPV16 accounted for the highest number of ICC cases in all age groups. The number of cases attributable to HPV16 and HPV18 was highest in the age group 35–44 years and decreased by age. The same pattern was observed for HPV31/33/45. The number of cases attributable to HPV52/58, HPV35/39/51/56/59, probably/possibly high‐risk types and low‐risk types remained low in all age groups. The highest PA of HPV16 was observed among cases aged 25–34 years (63.9%, 95% CI: 58.3–69.7), ranging from 41.7% to 62.6% in the other age groups (Figure 2D and Table S9). The highest PA of HPV18 was observed among cases aged 35–44 years (25.5%, 95% CI: 21.0–29.9), ranging from 9.2% to 25.0% in the other age groups. The cumulative PA of HPV16 and HPV18 was highest in age 25–34 years (85.9%, 95% CI: 81.4–90.1) and lowest in cases 75 years or older (60.0%, 95% CI: 49.7–70.5). Across age groups, the PA of HPV31/33/45 ranged from 11.9% to 25.0%, with overlapping 95% CIs. The highest PA of HPV52/58 was observed with 5 cases in the age group 75 years or older (PA = 5.9%, 95% CI: 1.2–11.6). For HPV35/39/51/56/59, the highest PA was observed in the age group 75 years or older (10.6%, 95% CI: 5.1–17.7). Excluding vaccinated ICC cases had only minor impact on the PAs of genotypes.

### 3.3 Control Section Assessment

Among HPV positive cases reported during 2017–2021, histopathological diagnoses were confirmed in control sections for 95.4% of CIN2/3 cases, 77.0% of AIS cases and 92.9% of ICC cases, with the majority of confirmations occurring in both control sections (Table S10). Most of the unconfirmed CIN2/3 cases showed less advanced morphology in control sections. Among unconfirmed AIS cases, most control sections showed CIN2/3 or less advanced morphology.

The CPs of HPV16, HPV31, HPV45, HPV51, HPV52 and HPV58 were higher in confirmed than in unconfirmed CIN2/3 cases (Table S11). Conversely, the high‐risk types HPV39 and HPV56 and all probably/possibly high‐risk types were more prevalent in unconfirmed cases. Among AIS cases, the CPs of HPV16, HPV18 and HPV45 were higher in confirmed than unconfirmed cases, while lower prevalences of HPV31, HPV33, HPV39, HPV51, HPV52, HPV56 and HPV58 were observed in confirmed cases. In ICCs, the genotype prevalences were similar in confirmed and unconfirmed cases.

When unconfirmed cases were included, the PAs were similar to what was observed among the confirmed cases, with a few exceptions for AIS cases where the PA of HPV18 decreased from 54.9% in confirmed specimens to 49.5% in all specimens (Table S12). The PA of HPV31 increased from 0.9% in confirmed cases to 3.4% in all cases, but remained the fourth most frequent genotype in AIS.

### 3.4 Vaccine Impact Population

During 2017–2024, 9379 cases were selected for HPV genotyping (Figure 1B). Of these, 2848 were born in 1991 or later and thus eligible for HPV immunisation programmes in Norway. 230 vaccine‐eligible cases were excluded because their specimen was not genotyped. Of the excluded cases, 167 were awaiting genotyping at the time of data extraction (of which 165 cases were reported in 2024). 2560 cases (97.8%) tested positive for at least one HPV genotype. After exclusion of unconfirmed cases, 1695 CIN2/3 cases, 470 AIS cases and 140 ICC cases were included in the vaccine impact population.

Among those included in the vaccine impact assessment, 154 of CIN2/3, 25 of AIS and 5 of ICC cases were routine‐eligible; the remaining were catch‐up‐eligible (Table 1). The proportion of unvaccinated cases ranged from 36.2% for AIS to 44.3% for ICC. Cases vaccinated before age 15 years included 98 CIN2/3, < 5 AIS and < 5 ICC cases. Most vaccinated cases had received three doses (Table S13).

### 3.5 Genotype Distribution by Vaccination Status in Catch‐Up Eligible Cases

The majority (94.9%) of vaccinated catch‐up‐eligible cases were vaccinated with 2vHPV (data not shown). In catch‐up‐eligible CIN2/3 cases, the seven types with the highest PA were the same in both vaccinated and unvaccinated women (in descending order): HPV16, HPV31, HPV33, HPV52, HPV18, HPV58 and HPV51 (Figure 3 and Table S14). The PA of HPV16 among catch‐up‐eligible CIN2/3 cases was lower in those vaccinated (35.5%, 95% CI: 32.5–38.6) versus those unvaccinated (45.7%, 95% CI: 41.9–49.7). The PA of HPV18, however, was similar in vaccinated (5.8%, 95% CI: 4.5–7.3) and unvaccinated cases (5.6%, 95% CI: 4.1–7.3). Among catch‐up‐eligible AIS cases, HPV18, HPV16 and HPV45 were the three types with the largest PA in both vaccinated and unvaccinated cases. In catch‐up‐eligible ICCs, the PAs of HPV16 was 62.5% (95% CI: 50.3–73.4) among unvaccinated versus 63.0% (95% CI: 52.2–74.0) in vaccinated cases, while the PAs of HPV18 was 15.8% (95% CI: 7.6–25.7) in unvaccinated and 19.2% (95% CI: 10.8–28.6) in vaccinated cases.

**FIGURE 3:**
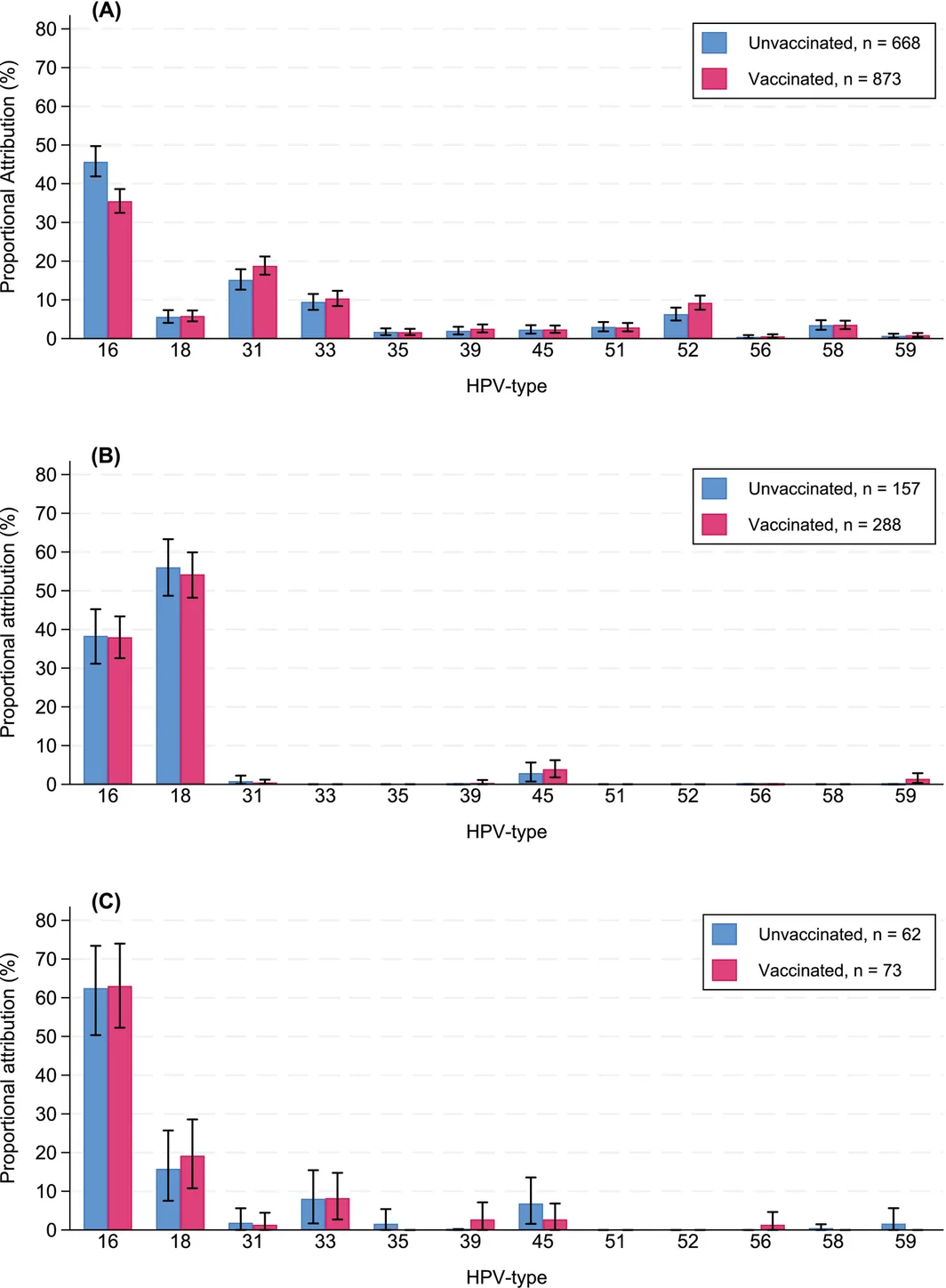
Proportional attribution with 95% confidence intervals of high‐risk HPV genotypes among catch‐up‐eligible cases (born 1991–1996) reported during 2017–2024. (A) For cervical intraepithelial neoplasia grade 2 or 3 (CIN2/3). (B) For adenocarcinoma in situ (AIS). (C) For invasive cervical cancer (ICC). Unvaccinated: Not registered with any HPV vaccine doses or the first dose received 365 days or less before they were first diagnosed with CIN2/3, AIS or ICC. Vaccinated: Received at least one dose of HPV vaccine more than 365 days before they were first diagnosed with CIN2/3, AIS or ICC.

### 3.6 Genotype Distribution by Vaccination Status in Routine‐Eligible Cases

Among 35 routine‐eligible unvaccinated cases of CIN2/3, most were attributed to HPV16 or HPV52 (PA of 49.9% (95% CI: 33.7–66.1) and 14.5% (95% CI: 5.1–26.9), respectively) (Figure 4 and Table S15). For routine‐eligible cases vaccinated before age 15 years (*n* = 95, all vaccinated with 4vHPV), HPV16 nearly disappeared (1 case), no cases were attributed to HPV18, and cases were mostly attributed to other high‐risk genotypes (in descending order: HPV58, HPV33, HPV52, HPV51 or HPV31). Among the routine‐eligible CIN2/3 cases vaccinated at age 15 years or later, the PA of HPV16 was also considerably lower (29.2%, 95% CI: 12.5–48.1) compared with unvaccinated cases (49.9%, 95% CI: 33.7–66.1). All routine‐eligible CIN2/3 cases vaccinated at age 15 years or later were vaccinated with 2vHPV and one case had been vaccinated with both 2vHPV and 9vHPV (data not shown).

**FIGURE 4:**
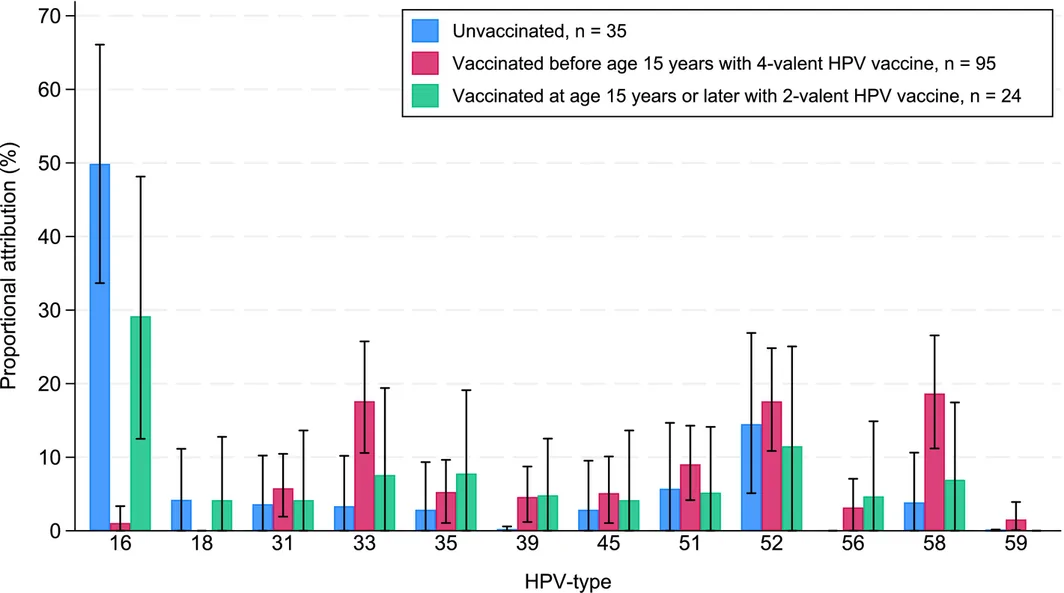
Proportional attributions with 95% confidence intervals of high‐risk human papillomavirus (HPV) genotypes among routine‐eligible women (born 1997 or later) with cervical intraepithelial neoplasia grade 2 or 3 (CIN2/3) reported during 2017–2024. Unvaccinated: Not registered with any HPV vaccine doses or the first dose received 365 days or less before they were first diagnosed with CIN2/3. Vaccinated before age 15 years: Received at least one dose of HPV vaccine 365 days or more before they were first diagnosed with CIN2/3 and prior to the year they turned 15 years old. All had received the 4‐valent HPV vaccine. Vaccinated at age 15 years or later: Received at least one dose of HPV vaccine 365 days or more before they were first diagnosed with CIN2/3, with the first dose administered the year they turned 15 years or later. All had received the 2‐valent HPV vaccine.

## 4 Discussion

This study is the first to present data from the mandatory national surveillance system on HPV genotypes in histopathological specimens in Norway, established in 2017. In samples from predominantly unvaccinated cases reported during 2017–2021, we found that three‐quarters of ICC cases were attributable to HPV16 or HPV18, followed by HPV45, HPV33 and HPV31. HPV16 was also the most common genotype in CIN2/3 lesions, followed by HPV31, HPV33, HPV52, HPV18 and HPV58. HPV16 was the most important genotype in both CIN2/3 and ICC, overall and in all age groups. About half of CIN2/3 cases were attributable to HPV16 or HPV18, and almost all AIS cases were attributable to HPV18, HPV16 or HPV45. Vaccine impact was assessed in cases reported during 2017–2024. Among CIN2/3 cases that were eligible for vaccination at age 11–12 years, almost half of the unvaccinated cases were attributed to HPV16, whereas HPV16 had nearly disappeared in those vaccinated before age 15 years. Among women with CIN2/3 that were eligible for HPV catch‐up vaccination, the attribution of HPV16 was also lower among vaccinated than unvaccinated cases. Only minor differences could be observed between unvaccinated and vaccinated catch‐up‐eligible cases of AIS and ICC.

A major strength of our study is the population‐based design and the representativeness of the cases, as we included practically all cancer cases, a random sample of all CIN2/3 and all AIS cases younger than 32 years in Norway. Furthermore, our study is the largest study on genotypes in AIS to date. In addition, due to the rigorous testing for 37 HPV types, the HPV detection rate was high, and the proportion of HPV negative cases was low compared to many other studies. Finally, the histopathological review is important because different lesions with varying morphologies in close proximity within the cervix are often caused by different genotypes and the PCR‐tested material may not match the histopathological diagnosis [22, 28]. In samples where the histopathological diagnosis was confirmed in at least one control section, HPV genotypes were more representative of types known to be associated with that histopathological diagnosis.

Our study also had some limitations. First, AIS cases above age 32 years were only included until October 2018, limiting our ability to assess impact of vaccination on genotypes in older age groups. Second, the baseline population included some vaccinated cases, since the surveillance started after vaccine introduction. The overall impact of excluding vaccinated cases was minimal, though minor changes in PAs were noted in CIN2/3 cases under age 25 years. We also assume the impact of herd immunity is minimal, as most unvaccinated vaccine‐eligible cases were from the catch‐up‐eligible cohorts, among whom we observed a smaller vaccine impact. Cases were also too old to have been impacted by boys' vaccination that started in 2018. Third, we assessed genotype distribution among cases only, so we could not estimate vaccine impact on the population‐level incidence. Lastly, due to coding practices, we could not distinguish between CIN2 and CIN3 [29], limiting comparability to other studies.

We have calculated the PAs of genotypes in cervical lesions as they more accurately represent causative genotypes than the CPs [30]. However, the differences between the CPs and the PAs were minor, and the choice of method did not impact the interpretation of our results.

Our estimates on genotypes in ICC align with a recent meta‐analysis by Wei et al., where HPV16/18 accounted for about 75% of cases, HPV31/33/45/52/58 for an additional 15%–20%, and the remaining HPV‐types judged as carcinogenic in their analysis (HPV35/59/39/56/51/68/73/26/69/82) were responsible for approximately 5% across all global regions [5]. HPV16 was the predominant genotype in our study, followed by HPV18, consistent with global and European data [5, 31, 32, 33]. The order of genotypes in ICC following HPV18 (HPV45, HPV33, HPV31 and HPV52), aligns with findings in the European sub‐analysis in Wei et al. [5]. More surprisingly, HPV73 ranked seventh in our study. Although HPV73‐associated ICC is rare in Norway, its carcinogenic potential has recently been recognised and further surveillance is warranted [5, 34]. Few ICC cases in our study were attributable to low‐risk genotypes. Although low‐risk HPV types have occasionally been reported as single infections in ICC [31], their causal role remains uncertain. For some ICCs positive on low‐risk types only, the true causative HPV type may not have been detected, either because of loss of target DNA, often occurring during integration of viral DNA into the host genome, or due to insufficient sensitivity of the genotyping assay [35]. The same may be true for HPV negative cases. Previous studies have shown that among HPV PCR‐negative ICC samples, subsequent testing using next‐generation sequencing (NGS) can detect HPV DNA, including genotypes targeted by the initial PCR assay [36]. To increase testing sensitivity, we re‐tested Luminex negative samples sequentially according to a fixed protocol and identified additional HPV genotype‐positive cases using Anyplex/Allplex or E6/E7. With NGS testing of Luminex‐negative samples, even more HPV genotype infections may have been detected, but NGS was not feasible within our surveillance system at the time of implementation.

The HPV16/18 CP of 49.8% (95% CI: 48.0–51.5) among baseline CIN2/3 cases in our study aligns with the 52% in a global meta‐analysis by Smith et al. [33]. HPV16 was the predominant genotype in CIN2/3 cases in the baseline population, consistent with global and European data [32, 33, 37]. However, the CP of HPV16 of 42.2% (95% CI: 40.5–44.0) in our study was slightly lower than previous global, European and Nordic estimates of 50%–60% [37, 38, 39]. One possible explanation for the lower prevalence of HPV16 in our study may be a different CIN2 to CIN3 ratio in our data, which we could not assess. We found that the five most common types following HPV16 in CIN2/3 cases were HPV31, HPV33, HPV52, HPV18 and HPV58. This closely aligns with the findings in a European multicentre study, except for HPV31 being less frequent and ranking third after HPV33 in their study [37]. Importantly, the prevalences of specific genotypes may not be directly comparable across studies due to differences in cervical screening practices, diagnostic criteria, age distribution of cases and PCR‐based HPV detection methods.

Few studies have examined the HPV type distribution in AIS. In line with previous studies [28, 32, 40, 41, 42, 43], HPV18 was far more common in AIS than in CIN2/3 in the baseline population, and most AIS lesions could be attributed to HPV18 or HPV16 and the remaining to HPV45. In agreement with this, most ACs in our study were attributed to these same three types, as corroborated by other research [31, 44]. Given the direct protection against HPV16/18 and the estimated cross‐protective vaccine effectiveness of about 83% against HPV45 from 2vHPV [26], it is expected that there will be very few cases of AIS and ACs in vaccinated young women in Norway in the future.

We observed that ICCs and CIN2/3 s were largely associated with the same HPV types. For both morphologies, the five types HPV16, HPV18, HPV31, HPV33 and HPV52 were ranked among the six most prevalent, although their order and PAs differed. HPV18 and HPV16 were more prevalent in ICC than in CIN2/3. HPV45 was also notably more prevalent in ICC compared to CIN2/3, as also observed in a meta‐analysis by Guan et al. [38], suggesting a higher carcinogenic potential of HPV45 compared to other non‐HPV16/18 types.

We estimated that the highest disease burden was in women aged 25–34 years for CIN2/3 and 35–44 years for ICC. We observed the highest cumulative PA of HPV16/18 in the age group 25–34 years for both CIN2/3 and ICC. This is in line with studies showing cervical lesions caused by HPV16/18 occur at a younger age compared to other types [37, 45]. Previous studies have reported a decrease of HPV16‐, HPV18‐ and HPV45‐attribution and a relative increase of HPV31, HPV33, HPV52 and HPV58 in ICC in older age [46]. Although the proportion of cases attributed to HPV16 and HPV18 was highest in younger age groups for both CIN2/3 and ICC in our study, their PAs did not consistently decrease with increasing age and HPV16 remained the most important genotype across all ages. In age groups where HPV16/18‐attribution was lower, the PAs of other genotypes necessarily increased, but no clear pattern of increase in specific HPV type groups was observed. While the highest PAs of the genotypes HPV52/58 and HPV35/39/51/56/59 were observed in ICC cases aged 75 years or older, the number of cases caused by these types remained low.

In our study, HPV16/18 nearly disappeared from CIN2/3 lesions in routine‐eligible cases vaccinated before age 15 years, demonstrating the effect of the 4‐valent vaccine and adding to the growing evidence of the HPV vaccine impact in Norway [27, 47, 48]. In CIN2/3 cases among catch‐up‐eligible women, there was a modest impact of vaccination. In line with a study that found that the prevalence of HPV infection among Norwegian 21‐year‐old women was 45.4% [49], our results indicate that many catch‐up‐eligible women may have been exposed to HPV infection prior to vaccination. This does not mean there was no impact of the catch‐up vaccination programme in Norway. We could not assess the impact on incidences in our data, but a study in Northern Norway has demonstrated a reduced incidence of HPV16/18‐induced lesions in the catch‐up population after vaccination [47].

As HPV16/18 decreased in CIN2/3 lesions in routine‐vaccinated women, the PA of other genotypes necessarily increased since the total PA across all types adds up to 100%. While the five genotypes attributing the most to CIN2/3 in routine‐eligible but unvaccinated women were HPV16, HPV52, HPV51, HPV18 and HPV58, the five types attributing the most to routine‐eligible CIN2/3 cases vaccinated before age 15 years were HPV58, HPV52, HPV33, HPV51 and HPV35. The latter five types are typically more prevalent in CIN2/3 than in ICC and have a lower propensity to develop to malignancy compared to HPV16/18 [38]. As pointed out by Cuschieri et al., in the vaccine‐era, a larger proportion of cervical lesions will be caused by non‐vaccine types that carry a lower risk of progression to ICC [50]. They suggest more research into what constitutes high‐grade disease in vaccinated populations and potentially establishing additional biomarkers. Our findings cannot be interpreted as an increased risk of CIN2/3 from non‐HPV16/18 types in vaccinated women, as we have studied HPV type proportions rather than rates in the population. The genotypes predominately associated with CIN2/3 among vaccinated women have a lower risk of progressing to ICC, and the HPV primary screening test has a lower positive predictive value among vaccinated, which is important to consider in the design of the screening programme in Norway.

## 5 Conclusion

In conclusion, the national surveillance system is well suited for HPV genotype surveillance in the vaccine era in Norway. The baseline genotype distribution in ICC and high‐grade cervical lesions in Norway aligns with European and international data. HPV16 was the most important genotype in both CIN2/3 and ICC, overall and in all age groups. HPV16/18 caused 74.6% (95% CI: 72.1–77.1) of ICC before vaccine impact. In CIN2/3 cases vaccinated prior to age 15 years, HPV16/18 had nearly disappeared. These results indicate strong protection against HPV16/18‐induced CIN2/3 in cohorts of women immunised prior to age 15 years.

## Author Contributions

**Terese Bekkevold:** conceptualization, data curation, formal analysis, investigation, methodology, project administration, writing – original draft, writing – review and editing. **Ida Laake:** conceptualization, formal analysis, methodology, validation, writing – review and editing. **Berit Feiring:** conceptualization, methodology, validation, writing – review and editing. **Kristiane Søreng:** methodology, investigation, validation, writing – review and editing. **Gro Kummeneje Presthus:** methodology, investigation, validation, writing – review and editing. **Glenny Cecilie Alfsen:** methodology, investigation, validation, writing – review and editing. **Irene Kraus Christiansen:** conceptualization, investigation, methodology, validation, writing – review and editing. **Mona Hansen:** investigation, methodology, validation, writing – review and editing. **Hanne Kristiansen:** investigation, methodology, validation, writing – review and editing. **Eirik Olsen:** data curation, writing – review and editing. **Torstein Gjølgali Frengen:** methodology, validation, writing – review and editing. **Emily A. Burger:** methodology, validation, writing – review and editing. **Christine Genieve Monceyron Jonassen:** conceptualization, methodology, validation, writing – review and editing. **Lill Trogstad:** conceptualization, project administration, methodology, validation, writing – review and editing, supervision.

## Ethics Statement

Ethical approval was not required for this work because the data analyses are conducted within the scope of rights afforded to the NIPH. The processing of data is authorised under the provisions § 7–9 of the Act relating to control of communicable diseases (LOV‐1994‐08‐05‐55), §§11 and 19 c in the Personal Health Data Filing System Act (LOV‐2014‐06‐20‐43), and within the scopes of the MSIS‐regulation (FOR‐2003‐06‐20‐740) and the SYSVAK‐regulation (FOR‐2003‐06‐20‐739), as described in §§ 1–3 and 1–7. Together, these provisions permit the processing of health data without individual consent when necessary to fulfil the purposes of the registers.

## Conflicts of Interest

The authors declare no conflicts of interest.

## Supporting information


**Table S1:** Information on primers used in the E6E7 type‐specific real‐time PCR.
**Table S2:** PCR conditions used for the E6E7 type‐specific real‐time PCR.
**Table S3:** Variables from the Norwegian Surveillance System for Communicable Diseases (MSIS) included in the study.
**Table S4:** Number of HPV vaccinated cases by age group in the baseline population reported during 2017–2021.
**Table S5:** Crude prevalence and proportional attribution of HPV genotypes by sub‐morphology of invasive cervical cancer in the baseline population reported during 2017–2021.
**Table S6:** Crude prevalence of HPV genotypes by morphology in the baseline population (2017–2021), with results shown separately for Luminex‐positive samples, Anyplex/Allplex‐positive samples following a negative Luminex result, and in‐house E6/E7‐positive samples following negative Luminex and Anyplex/Allplex results.
**Table S7:** Crude prevalence among single infected cases and hierarchical prevalence of HPV genotypes by morphology in the baseline population reported during 2017–2021.
**Table S8:** Proportional attribution of HPV genotypes for all cases and unvaccinated cases of CIN2/3, by age group.
**Table S9:** Proportional attribution of HPV genotypes in all cases and unvaccinated cases of ICC by age groups.
**Table S10:** Morphology of control sections of all genotyped samples from cases reported during 2017–2021.
**Table S11:** Crude prevalence of HPV genotypes in cases reported during 2017–2021, with histopathologically confirmed control section(s) versus unconfirmed cases.
**Table S12:** Proportional attribution of HPV genotypes in cases reported during 2017–2021, including specimens where histopathological diagnosis is not confirmed in either control sections.
**Table S13:** Human papillomavirus (HPV) vaccination status in vaccine‐eligible cases (born 1991 or later) reported during 2017–2024, by morphology and birth cohort.
**Table S14:** Proportional attribution of HPV genotypes in catch‐up‐eligible cases (born 1991–1996) reported during 2017–2024, by morphology and vaccination status.
**Table S15:** Proportional attribution of HPV genotypes by vaccination status in routine‐eligible cases (born 1997 or later) with cervical intraepithelial neoplasia grade 2 or 3 (CIN2/3), reported during 2017–2024.

## Data Availability

We have used personal data from the Norwegian Surveillance System for Communicable Diseases and the Norwegian Immunisation Registry in this study. Access to data requires an application to www.helsedata.no. Further information is available from the corresponding author upon request.
