# Supplementary material for: HPV Genotype Distribution in Cervical Cancer and High‐Grade Lesions in Norway, and Early Vaccine Impact
Source: Int J Cancer. 2026 Aug 28;160(1):171–86. doi: 10.1002/ijc.70712 (PMC13729978; doi:10.1002/ijc.70712)
Supplement: Supplementary file 1 [file IJC-160-171-s001.pdf]

Supplementary material:

HPV genotype distribution in cervical cancer and high-grade lesions in Norway, and early vaccine impact

Terese Bekkevold, Ida Laake, Berit Feiring, Kristiane Søreng, Gro Kummeneje Presthus, Glenny Cecilie Alfsen, Irene Kraus Christiansen, Mona Hansen, Hanne Kristiansen, Eirik Olsen, Torstein Gjølgali Frengen, Emily A. Burger, Christine Genieve Monceyron Jonassen, Lill Trogstad

Table of contents

Table S1: Information on primers used in the E6E7 type-specific real-time PCR.............................. 3

Table S2: PCR conditions used for the E6E7 type-specific real-time PCR... 5

Table S3: Variables from the Norwegian Surveillance System for Communicable Diseases (MSIS) included in the study. 6

Table S4: Number of HPV vaccinated cases by age group in the baseline population reported during 2017–2021. 9

Table S5: Crude prevalence and proportional attribution of HPV genotypes by sub-morphology of invasive cervical cancer in the baseline population reported during 2017–2021. 10

Table S6: Crude prevalence of HPV genotypes by morphology in the baseline population (2017–2021), with results shown separately for Luminex-positive samples, Anyplex/Allplex-positive samples following a negative Luminex result, and in-house E6/E7-positive samples following negative Luminex and Anyplex/Allplex results. 13

Table S7: Crude prevalence among single infected cases and hierarchical prevalence of HPV genotypes by morphology in the baseline population reported during 2017–2021....................... 15

Table S8: Proportional attribution of HPV genotypes for all cases and unvaccinated cases of CIN2/3, by age group.. 17

Table S9: Proportional attribution of HPV genotypes in all cases and unvaccinated cases of ICC by age groups. 19

Table S10: Morphology of control sections[1] of all genotyped samples from cases reported during 2017—2021. 22

Table S11: Crude prevalence of HPV genotypes in cases reported during 2017—2021, with histopathologically confirmed control section(s) versus unconfirmed cases. 23

Table S12: Proportional attribution of HPV genotypes in cases reported during 2017—2021, including specimens where histopathological diagnosis is not confirmed in either control sections[1]. 25

Table S13: Human papillomavirus (HPV) vaccination status in vaccine-eligible cases (born 1991 or later) reported during 2017—2024, by morphology and birth cohort. 27

Table S14: Proportional attribution of HPV genotypes in catch-up-eligible cases (born 1991—1996) reported during 2017—2024[1], by morphology and vaccination status. 28

Table S15: Proportional attribution of HPV genotypes by vaccination status in routine-eligible cases (born 1997 or later) with cervical intraepithelial neoplasia grade 2—3 (CIN2/3), reported during 2017—2024[1]. 32

Table S1: Information on primers used in the E6E7 type-specific real-time PCR

Target	Reference	Primer direction	Sequence	Annealing temperature	Product length
HPV16	Karlsen et al[1]	Forward	TCAAAAGCCACTGTGTCCTGA	57°C	120 bp
		Reverse	CGTGTTCTTGATGATCTGCAA		
HPV18	Karlsen et al[1]	Forward	CCGAGCACGACAGGAACGACT	57°C	173 bp
		Reverse	TCGTTTTCTTCCTCTGAGTCGCTT		
HPV31	Lindh et al[2]	Forward	ATTCCACAACATAGGAGGAAGGTG	55°C	76 bp
		Reverse	CACTTGGGTTTCAGTACGAGGTCT		
HPV33	Lindh et al[2]	Forward	ATATTTCGGGTCGTTGGGCA	57°C	74 bp
		Reverse	ACGTCACAGTGCAGTTTCTCTACGT		
HPV35	Karlsen et al[1]	Forward	CCCGAGGCAACTGACCTATA	57°C	231 bp
		Reverse	GGGGCACACTATTCCAAATG		
HPV39	Lindh et al[2]	Forward	GCAGGAAGCTATACAGGACAGTGTC	54°C	82 bp
		Reverse	CTTGGGTTTCTCTTCGTGTTAGTCT		
HPV45	Dictor et al[3]	Forward	GGACAGTACCGAGGGCAGTGTA	58°C	107 bp
		Reverse	CCGGGGTCCATGCATACTTAT		
HPV51	Lindh et al[2]	Forward	AAAGCAAAAATTGGTGGACGA	54°C	81 bp
		Reverse	TGCCAGCAATTAGCGCATT		
HPV52	Dictor et al[3]	Forward	GTTGGACAGGGCGCTGTTC	58°C	166 bp
		Reverse	CCTCCTCATCTGAGCTGTCACC		
HPV56	Lindh et al[2]	Forward	TGCATTGTGACAGAAAAAGACGAT	54°C	73 bp
		Reverse	CTCCAGCACCCCAAACATG		
HPV58	Dictor et al[3]	Forward	AGGGCGCTGTGCAGTGTGT	58°C	172 bp
		Reverse	CATCCTCGTCTGAGCTGTCACA		
HPV59	Sotlar et al[4]	Forward	CAAAGGGGAACTGCAAGAAAG	54°C	195 bp
		Reverse	TATAACAGCGTATCAGCAGC		
HPV66	Sotlar et al[4]	Forward	TTCAGTGTATGGGGCAACAT	56°C	172 bp
		Reverse	AAACATGACCCGGTCCATGC		

Target	Reference	Primer direction	Sequence	Annealing temperature	Product length
HPV68	In-house	Forward	TCACGAGCAATTAGRAGATT	54°C	
	In-house	Reverse	GTCTGGCTAGTAGTWGATGT		

[1]Karlsen F, Kalantari M, Jenkins A, Pettersen E, Kristensen G, Holm R, et al. Use of multiple PCR primer sets for optimal detection of human papillomavirus. J Clin Microbiol. 1996;34(9):2095-100.
[2]Lindh M, Görander S, Andersson E, Horal P, Mattsby-Balzer I, Ryd W. Real-time Taqman PCR targeting 14 human papilloma virus types. J Clin Virol. 2007;40(4):321-4.
[3]Dictor M, Warenholt J. Single-tube multiplex PCR using type-specific E6/E7 primers and capillary electrophoresis genotypes 21 human papillomaviruses in neoplasia. Infect Agent Cancer. 2011;6(1):1.
[4] Sotlar K, Diemer D, Dethleffs A, Hack Y, Stubner A, Vollmer N, et al. Detection and typing of human papillomavirus by e6 nested multiplex PCR. J Clin Microbiol. 2004;42(7):3176-84.

Table S2: PCR conditions used for the E6E7 type-specific real-time PCR.

Step	Temperature	Duration	Cycles
UDG Activation	50°C	2 min	Hold
AmpliTaq®DNA Polymerase, UP Activation	95°C	2 min	Hold
Denaturation	95°C	15 sec	40
Anneal	55-60°C[1]	15 sec	
Extend	72°C	1 min	

[1]The specific annealing temperatures are shown in Table S1.

Table S3: Variables from the Norwegian Surveillance System for Communicable Diseases (MSIS) included in the study.

Original source	Variable name	Description	Values
MSIS	TilfelleId	Registry internal identifier, unique for each disease episode (case)	
MSIS	PasientNr	Registry internal identifier, unique for each person	
MSIS	PatologisvarId	Registry internal identifier, unique for the histopathological specimen	
MSIS	HpvsvarId	Registry internal identifier, unique for each HPV test performed	
MSIS	AnalysesvarId	Registry internal identifier, unique for each HPV genotype result	
KRG[1]	TilfelleOpprettetTidspunkt	Date and time the case was reported from KRG	
KRG[1]	Fodelsar	Birthyear	
KRG[1]	Diaganosear	Year the case was first diagnosed	
KRG[1]	Provetakningsdato	Date the histopathological specimen was retrieved from the cervix	
KRG[1]	Diagnosedato	Diagnosis date, specific for each histological sample.	
KRG[1]	HovedmorfologiKategoriBeskrivelse	The morphology category related to the specific histological sample	CIN2/3<br>AIS<br>Squamous cell carcinoma<br>Adenocarcinoma

Original source	Variable name	Description	Values
			Other carcinoma
Reflab[2]	HpvKonklusjonBeskrivelse	Conclusion from human papillomavirus genotyping	Positive<br>Negative<br>Inconclusive<br>Not analyzed: excluded<br>Not analyzed: changed or wrong diagnosis<br>Not analyzed: not representative material<br>Not analyzed: material not available<br>Not analyzed: sample do not exist
Reflab[2]	Analysekode	Human papillomavirus genotype	HPV6<br>HPV11<br>HPV16<br>HPV18<br>HPV26<br>HPV30<br>HPV31<br>HPV33<br>HPV35<br>HPV39<br>HPV40<br>HPV42<br>HPV43<br>HPV44<br>HPV45<br>HPV51<br>HPV52<br>HPV53<br>HPV54<br>HPV56<br>HPV58<br>HPV59<br>HPV61<br>HPV66<br>HPV67<br>HPV68<br>HPV69<br>HPV70<br>HPV73<br>HPV74

Original source	Variable name	Description	Values
			HPV81<br>HPV82<br>HPV83<br>HPV86<br>HPV87<br>HPV89<br>HPV90<br>HPV91
Reflab[2]	Profil	Human papillomavirus genotype test	HPV-Luminex<br>HPV-Allplex<br>HPV-Anyplex<br>HPV-E6E7
Reflab[2]	Svar	The result of the human papillomavirus genotype test	Not performed<br>Negative<br>Inconclusive<br>Positive<br>Weak positive result
Reflab[2]	Analysedato	Date the human papillomavirus genotype test was performed	
Reflab[2]	Kontrollsnitt1Diagnose	Morphology in control section[3] 1	HIST0: no HPV-induced lesions/not analyzed<br>HIST1: CIN1<br>HIST2: CIN2/3<br>HIST3: AIS<br>HIST4: ICC<br>IKKE UTF: not analyzed<br>TOM: empty block
Reflab[2]	Kontrollsnitt2Diagnose	Morphology in control section[3] 2	HIST0: no HPV-induced lesions/not analyzed<br>HIST1: CIN1<br>HIST2: CIN2/3<br>HIST3: AIS<br>HIST4: ICC<br>IKKE UTF: not analyzed<br>TOM: empty block

CIN2/3: Cervical intraepithelial neoplasia grade 2—3, AIS: Adenocarcinoma in situ, ICC: Invasive cervical cancer. [1]KRG: Cancer Registry of Norway. [2]Reflab: The national HPV Reference Laboratory at Akershus University Hospital. [3]Haematoxylin-eosin-stained sections adjacent to the genotyped section.

Table S4: Number of HPV vaccinated cases by age group in the baseline population reported during 2017–2021.

	CIN2/3<br>N = 3087	AIS<br>N = 550	ICC<br>N = 1265
	**n vaccinated[1] (%)**	**n vaccinated[1] (%)**	**n vaccinated[1] (%)**
All vaccinated[1] cases	473 (15.3)	175 (31.8)	44 (3.5)
**Age groups:**			
<25 years	76 (46.9)	13 (30.2)	6 (50.0)
25-34 years	381 (22.9)	162 (38.5)	38 (15.3)
35-44 years	11 (1.5)	0 (0)	NA**
45-54 years	5 (1.5)	0 (0)	0 (0)
55-64 years	NA*	0 (0)	0 (0)
65-74 years	0 (0)	0 (0)	0 (0)
≥75 years	0 (0)	0 (0)	0 (0)

CIN2/3: Cervical intraepithelial neoplasia grade 2—3, AIS: Adenocarcinoma in situ, ICC: Invasive cervical cancer.
[1]Vaccinated: received at least one dose of HPV vaccine 365 days or more before they were first diagnosed with CIN2/3, AIS or ICC.

* Since there were fewer than 5 vaccinated cases in the age group 55—64 years, they were anonymized by reporting them together with the age group 45—54 years.

** Since there were fewer than 5 vaccinated cases in the age group 35—44 years, they were anonymized by reporting them together with the age group 25—34 years.

Table S5: Crude prevalence and proportional attribution of HPV genotypes by sub-morphology of invasive cervical cancer in the baseline population reported during 2017–2021.

	Crude prevalence			Proportional attribution		
	SCC N = 975	*AC N = 221*	*Other ICC N = 69*	*SCC N = 975*	*AC N = 221*	*Other ICC N = 69*
	**n (%)**	**n (%)**	**n (%)**	**n[1] (%)**	**n[1] (%)**	**n[1] (%)**
**Number of HPV types**						
1 type	939 (96.3)	214 (96.8)	67 (97.1)	-	-	-
2 types	32 (3.3)	7 (3.2)	2 (2.9)	-	-	-
≥ 3 types	4 (0.4)	0 (0)	0 (0)	-	-	-
**High-risk HPV types**						
16	590 (60.5)	94 (42.5)	20 (29.0)	589.0 (60.4)	93.5 (42.3)	19.3 (28.0)
18	108 (11.1)	105 (47.5)	35 (50.7)	102.8 (10.5)	104.4 (47.2)	34.7 (50.2)
31	45 (4.6)	7 (3.2)	1 (1.4)	43.0 (4.4)	4.1 (1.9)	1.0 (1.5)
33	63 (6.5)	0 (0)	4 (5.8)	63.0 (6.5)	0 (0)	4.0 (5.8)
35	10 (1.0)	0 (0)	0 (0)	8.6 (0.9)	0 (0)	0 (0)
39	15 (1.5)	0 (0)	1 (1.4)	14.0 (1.4)	0 (0)	1.0 (1.5)
45	62 (6.4)	14 (6.3)	5 (7.2)	59.5 (6.1)	14.0 (6.3)	5.0 (7.2)
51	3 (0.3)	0 (0)	0 (0)	1.0 (0.1)	0 (0)	0 (0)
52	25 (2.6)	0 (0)	0 (0)	24.7 (2.5)	0 (0)	0 (0)
56	14 (1.4)	1 (0.5)	0 (0)	13.9 (1.4)	1.0 (0.4)	0 (0)
58	10 (1.0)	0 (0)	0 (0)	7.4 (0.8)	0 (0)	0 (0)
59	9 (0.9)	3 (1.4)	1 (1.4)	7.1 (0.7)	3.0 (1.4)	1.0 (1.5)
**Probably/possibly high-risk HPV types**						
26	0 (0)	0 (0)	0 (0)	0 (0)	0 (0)	0 (0)
30	3 (.3)	0 (0)	0 (0)	2.1 (0.2)	0 (0)	0 (0)
53	1 (.1)	0 (0)	0 (0)	0 (0)	0 (0)	0 (0)
66	1 (.1)	0 (0)	0 (0)	1.0 (0.1)	0 (0)	0 (0)
67	2 (0.2)	0 (0)	0 (0)	2.0 (0.2)	0 (0)	0 (0)
68	8 (0.8)	1 (0.5)	1 (1.4)	7.1 (0.7)	1.0 (0.4)	1.0 (1.5)
69	0 (0)	0 (0)	0 (0)	0 (0)	0 (0)	0 (0)
70	7 (0.7)	0 (0)	0 (0)	6.0 (0.6)	0 (0)	0 (0)

	Crude prevalence			Proportional attribution		
	SCC N = 975	***AC N = 221***	***Other ICC N = 69***	***SCC N = 975***	***AC N = 221***	***Other ICC N = 69***
	**n (%)**	**n (%)**	**n (%)**	**n[1] (%)**	**n[1] (%)**	**n[1] (%)**
73	19 (1.9)	0 (0)	1 (1.4)	17.6 (1.8)	0 (0)	1.0 (1.5)
82	10 (1.0)	0 (0)	1 (1.4)	1.2 (0.1)	0 (0)	0 (0)
**Low-risk HPV types**						
6	2 (0.2)	2 (0.9)	0 (0)	1.0 (0.1)	0 (0)	0 (0)
11	0 (0)	0 (0)	0 (0)	0 (0)	0 (0)	0 (0)
40	2 (0.2)	0 (0)	0 (0)	0 (0)	0 (0)	0 (0)
42	4 (0.4)	0 (0)	0 (0)	3.1 (0.3)	0 (0)	0 (0)
43	0 (0)	1 (.5)	0 (0)	0 (0)	0 (0)	0 (0)
54	0 (0)	0 (0)	0 (0)	0 (0)	0 (0)	0 (0)
61	0 (0)	0 (0)	0 (0)	0 (0)	0 (0)	0 (0)
74	0 (0)	0 (0)	0 (0)	0 (0)	0 (0)	0 (0)
81	0 (0)	0 (0)	0 (0)	0 (0)	0 (0)	0 (0)
83	0 (0)	0 (0)	0 (0)	0 (0)	0 (0)	0 (0)
86	0 (0)	0 (0)	0 (0)	0 (0)	0 (0)	0 (0)
87	1 (0.1)	0 (0)	0 (0)	0 (0)	0 (0)	0 (0)
89	0 (0)	0 (0)	0 (0)	0 (0)	0 (0)	0 (0)
90	0 (0)	0 (0)	0 (0)	0 (0)	0 (0)	0 (0)
91	1 (0.1)	0 (0)	1 (1.4)	0 (0)	0 (0)	1.0 (1.5)
**Groups of high-risk HPV types**						
16/18	693 (71.1)	198 (89.6)	54 (78.3)	692.1 (71.0)	197.5 (89.4)	54.0 (78.3)
31/33/45	170 (17.4)	21 (9.5)	10 (14.5)	165.3 (17.0)	18.5 (8.4)	10.0 (14.5)
52/58	35 (3.6)	0 (0)	0 (0)	44.6 (4.6)	4.0 (1.8)	2.0 (2.9)
35/39/51/56/59	51 (5.2)	4 (1.8)	2 (2.9)	32.0 (3.3)	0 (0)	0 (0)
**HPV types grouped by risk category**						
High risk	936 (96.0)	220 (99.5)	66 (95.7)	934.1 (95.8)	220.0 (99.5)	66.0 (95.7)
Probably/possibly high-risk	51 (5.2)	1 (0.5)	3 (4.3)	36.9 (3.8)	1.0 (0.4)	2.0 (2.9)
Low-risk	10 (1.0)	3 (1.4)	1 (1.4)	4.1 (0.4)	0 (0)	1.0 (1.5)

SCC: Squamous cell carcinoma, AC: adenocarcinoma, Other ICC: invasive cervical cancer of unspecified etiology.

[1]Numbers are not whole due to weighting.

Table S6: Crude prevalence of HPV genotypes by morphology in the baseline population (2017–2021), with results shown separately for Luminex-positive samples, Anyplex/Allplex-positive samples following a negative Luminex result, and in-house E6/E7-positive samples following negative Luminex and Anyplex/Allplex results.

	CIN2/3<br>n = 3087			AIS<br>n = 550			ICC<br>n = 1265		
	***Luminex*[1]**	***Anyplex /Allplex*[2]**	***E6/E7*[3]**	***Luminex*[1]**	***Anyplex /Allplex*[2]**	***E6/E7*[3]**	***Luminex*[1]**	***Anyplex /Allplex*[2]**	***E6/E7*[3]**
	**n (%)**	**n (%)**	**n (%)**	**n (%)**	**n (%)**	**n (%)**	**n (%)**	**n (%)**	**n (%)**
**High-risk HPV types**									
**16**	1268 (41.1)	34 (1.1)	1 (0.03)	220 (40.0)	4 (0.7)	0 (0)	689 (54.5)	14 (1.1)	1 (0.08)
**18**	255 (8.3)	10 (0.3)	5 (0.2)	312 (56.7)	8 (1.5)	1 (0.2)	234 (18.5)	12 (0.9)	2 (0.2)
**31**	529 (17.1)	26 (0.8)	3 (0.10)	32 (5.8)	0 (0)	0 (0)	52 (4.1)	1 (0.08)	0 (0)
**33**	257 (8.3)	43 (1.4)	4 (0.1)	7 (1.3)	0 (0)	0 (0)	63 (5.0)	4 (0.3)	0 (0)
**35**	93 (3.0)	2 (0.06)	0 (0)	3 (0.5)	0 (0)	0 (0)	10 (0.8)	0 (0)	0 (0)
**39**	54 (1.7)	20 (0.6)	5 (0.2)	5 (0.9)	1 (0.2)	0 (0)	11 (0.9)	5 (0.4)	0 (0)
**45**	113 (3.7)	13 (0.4)	6 (0.2)	34 (6.2)	1 (0.2)	2 (0.4)	73 (5.8)	3 (0.2)	5 (0.4)
**51**	112 (3.6)	11 (0.4)	0 (0)	5 (0.9)	0 (0)	0 (0)	3 (0.2)	0 (0)	0 (0)
**52**	285 (9.2)	11 (0.4)	3 (0.10)	11 (2.0)	0 (0)	0 (0)	25 (2.0)	0 (0)	0 (0)
**56**	42 (1.4)	15 (0.5)	0 (0)	4 (0.7)	0 (0)	0 (0)	11 (0.9)	2 (0.2)	2 (0.2)
**58**	125 (4.0)	24 (0.8)	1 (0.03)	3 (0.5)	0 (0)	0 (0)	9 (0.7)	1 (0.08)	0 (0)
**59**	36 (1.2)	5 (0.2)	0 (0)	4 (0.7)	1 (0.2)	0 (0)	12 (0.9)	1 (0.08)	0 (0)
**Probably/possibly high-risk types**									
**26**	5 (0.2)	1 (0.03)	NA	0 (0)	0 (0)	NA	0 (0)	0 (0)	NA
**30**	16 (0.5)	NA	NA	0 (0)	NA	NA	3 (0.2)	NA	NA
**53**	25 (0.8)	13 (0.4)	NA	1 (0.2)	0 (0)	NA	1 (0.08)	0 (0)	NA
**67**	35 (1.1)	3 (0.10)	1 (0.03)	1 (0.2)	1 (0.2)	0 (0)	1 (0.08)	0 (0)	0 (0)
**66**	29 (0.9)	NA	NA	2 (0.4)	NA	NA	2 (0.2)	NA	NA
**68**	27 (0.9)	6 (0.2)	0 (0)	2 (0.4)	0 (0)	0 (0)	10 (0.8)	0 (0)	0 (0)
**69**	4 (0.1)	0 (0)	NA	0 (0)	0 (0)	NA	0 (0)	0 (0)	NA
**70**	26 (0.8)	1 (0.03)	NA	4 (0.7)	1 (0.2)	NA	7 (0.6)	0 (0)	NA
**73**	7 (0.2)	4 (0.1)	NA	0 (0)	0 (0)	NA	14 (1.1)	6 (0.5)	NA
**82**	30 (1.0)	26 (0.8)	NA	0 (0)	0 (0)	NA	10 (0.8)	1 (0.08)	NA

	CIN2/3<br>n = 3087			AIS<br>n = 550			ICC<br>n = 1265		
	***Luminex*[1]**	***Anyplex /Allplex*[2]**	***E6/E7*[3]**	***Luminex*[1]**	***Anyplex /Allplex*[2]**	***E6/E7*[3]**	***Luminex*[1]**	***Anyplex /Allplex*[2]**	***E6/E7*[3]**
	**n (%)**	**n (%)**	**n (%)**	**n (%)**	**n (%)**	**n (%)**	**n (%)**	**n (%)**	**n (%)**
**Low-risk types**									
**6**	26 (0.8)	2 (0.06)	NA	3 (0.5)	0 (0)	NA	4 (0.3)	0 (0)	NA
**11**	11 (0.4)	0 (0)	NA	1 (0.2)	0 (0)	NA	0 (0)	0 (0)	NA
**40**	4 (0.1)	0 (0)	NA	0 (0)	0 (0)	NA	2 (0.2)	0 (0)	NA
**42**	9 (0.3)	7 (0.2)	NA	1 (0.2)	0 (0)	NA	4 (0.3)	0 (0)	NA
**43**	8 (0.3)	1 (0.03)	NA	0 (0)	0 (0)	NA	0 (0)	1 (0.08)	NA
**44**	NA	2 (0.06)	NA	NA	0 (0)	NA	NA	0 (0)	NA
**54**	3 (0.10)	1 (0.03)	NA	0 (0)	0 (0)	NA	0 (0)	0 (0)	NA
**61**	1 (0.03)	2 (0.06)	NA	0 (0)	1 (0.2)	NA	0 (0)	0 (0)	NA
**74**	10 (0.3)	NA	NA	0 (0)	NA	NA	0 (0)	NA	NA
**81**	6 (0.2)	NA	NA	2 (0.4)	NA	NA	0 (0)	NA	NA
**83**	10 (0.3)	NA	NA	0 (0)	NA	NA	0 (0)	NA	NA
**86**	1 (0.03)	NA	NA	1 (0.2)	NA	NA	0 (0)	NA	NA
**87**	6 (0.2)	NA	NA	0 (0)	NA	NA	1 (0.08)	NA	NA
**89**	3 (0.10)	NA	NA	0 (0)	NA	NA	0 (0)	NA	NA
**90**	5 (0.2)	NA	NA	0 (0)	NA	NA	0 (0)	NA	NA
**91**	19 (0.6)	NA	NA	0 (0)	NA	NA	2 (0.2)	NA	NA

CIN2/3: Cervical intraepithelial neoplasia grade 2—3, AIS: Adenocarcinoma in situ, ICC: Invasive cervical cancer.

[1] A multiplex bead-based Luminex assay, which combines Modified General Primer PCR amplification with hybridization to HPV type-specific probes, detecting 37 HPV-types: HPV6/11/16/18/26/30/31/33/35/39/40/42/43/45/51/52/53/54/56/58/59/61/66/67/68/69/70/73/74/81/82/83/86/87/89/90/91.

[2] AnyplexTM II HPV28/AllplexTM HPV28 Detection from Seegene (Allplex replaced Anyplex in April 2024), detecting 28 HPV-types: HPV6/11/16/18/26/31/33/35/39/40/42/43/44/45/51/52/53/54/56/58/59/61/66/68/69/70/73/82.

[3]In-house type-specific real-time PCR targeting the E6E7 genes of the of 14 HPV types in the following order: HPV16, HPV18, HPV45, HPV31, HPV33, HPV52, HPV58, HPV35, HPV39, HPV51, HPV56, HPV59, HPV68 and HPV66.

Table S7: Crude prevalence among single infected cases and hierarchical prevalence of HPV genotypes by morphology in the baseline population reported during 2017–2021.

	Prevalence among single infected cases			Hierarchical prevalence[1]		
	CIN2/3 N= 2506	***AIS N = 431***	***ICC N = 1220***	***CIN2/3 N = 3087***	***AIS N = 550***	***ICC N = 1265***
	**n (%)**	**n (%)**	**n (%)**	**n (%)**	**n (%)**	**n (%)**
**High-risk HPV types**						
16	1068 (42.6)	160 (37.1)	682 (55.9)	1303 (42.2)	179 (32.5)	704 (55.7)
18	157 (6.3)	227 (52.7)	230 (18.9)	198 (6.4)	321 (58.4)	241 (19.1)
31	385 (15.4)	4 (0.9)	46 (3.8)	492 (15.9)	4 (0.7)	48 (3.8)
33	215 (8.6)	0 (0)	65 (5.3)	268 (8.7)	0 (0)	67 (5.3)
35	50 (2.0)	0 (0)	8 (0.7)	57 (1.8)	0 (0)	8 (0.6)
39	45 (1.8)	1 (0.2)	14 (1.1)	49 (1.6)	1 (0.2)	15 (1.2)
45	88 (3.5)	29 (6.7)	76 (6.2)	105 (3.4)	34 (6.2)	78 (6.2)
51	57 (2.3)	0 (0)	1 (0.1)	72 (2.3)	0 (0)	1 (0.1)
52	177 (7.1)	0 (0)	23 (1.9)	231 (7.5)	0 (0)	25 (2.0)
56	29 (1.2)	1 (0.2)	14 (1.1)	34 (1.1)	1 (0.2)	15 (1.2)
58	98 (3.9)	0 (0)	7 (0.6)	120 (3.9)	0 (0)	7 (0.6)
59	19 (0.8)	1 (0.2)	11 (0.9)	22 (0.7)	2 (0.4)	11 (0.9)
**Probably/possibly high-risk HPV types**						
26	2 (0.1)	0 (0)	0 (0)	2 (0.1)	0 (0)	0 (0)
30	4 (0.2)	0 (0)	2 (0.2)	4 (0.1)	0 (0)	2 (0.2)
53	15 (0.6)	0 (0)	0 (0)	17 (0.6)	0 (0)	0 (0)
66	16 (0.6)	1 (0.2)	1 (0.1)	19 (0.6)	1 (0.2)	1 (0.1)
67	10 (0.4)	0 (0)	2 (0.2)	10 (0.3)	0 (0)	2 (0.2)
68	12 (0.5)	1 (0.2)	9 (0.7)	13 (0.4)	1 (0.2)	9 (0.7)
69	1 (0.0)	0 (0)	0 (0)	1 (0.0)	0 (0)	0 (0)
70	11 (0.4)	3 (0.7)	6 (0.5)	13 (0.4)	3 (0.5)	6 (0.5)
73	4 (0.2)	0 (0)	17 (1.4)	5 (0.2)	0 (0)	19 (1.5)
82	21 (0.8)	0 (0)	1 (0.1)	28 (0.9)	0 (0)	1 (0.1)

	Prevalence among single infected cases			Hierarchical prevalence[1]		
	CIN2/3<br>*N= 2506*	*AIS*<br>*N = 431*	*ICC*<br>*N = 1220*	*CIN2/3*<br>*N = 3087*	*AIS*<br>*N = 550*	*ICC*<br>*N = 1265*
	**n (%)**	**n (%)**	**n (%)**	**n (%)**	**n (%)**	**n (%)**
**Low-risk HPV types**						
6	8 (0.3)	1 (0.2)	1 (0.1)	10 (0.3)	1 (0.2)	1 (0.1)
11	3 (0.1)	0 (0)	0 (0)	3 (0.1)	0 (0)	0 (0)
40	0 (0)	0 (0)	0 (0)	0 (0)	0 (0)	0 (0)
42	4 (0.2)	0 (0)	3 (0.2)	4 (0.1)	0 (0)	3 (0.2)
43	0 (0)	0 (0)	0 (0)	0 (0)	0 (0)	0 (0)
54	0 (0)	0 (0)	0 (0)	0 (0)	0 (0)	0 (0)
61	1 (0.0)	1 (0.2)	0 (0)	1 (0.0)	1 (0.2)	0 (0)
74	0 (0)	0 (0)	0 (0)	0 (0)	0 (0)	0 (0)
81	3 (0.1)	1 (0.2)	0 (0)	3 (0.1)	1 (0.2)	0 (0)
83	2 (0.1)	0 (0)	0 (0)	2 (0.1)	0 (0)	0 (0)
86	0 (0)	0 (0)	0 (0)	0 (0)	0 (0)	0 (0)
87	0 (0)	0 (0)	0 (0)	0 (0)	0 (0)	0 (0)
89	0 (0)	0 (0)	0 (0)	0 (0)	0 (0)	0 (0)
90	0 (0)	0 (0)	0 (0)	0 (0)	0 (0)	0 (0)
91	1 (0.0)	0 (0)	1 (0.1)	1 (0.0)	0 (0)	1 (0.1)

CIN2/3: cervical intraepithelial neoplasia grade 2 or 3. AIS: adenocarcinoma in situ. ICC: invasive cervical cancer.

[1]Hierarchical prevalence: in cases with multiple infections, only the most prevalent type for morphology contributes to the prevalence.

Table S8: Proportional attribution of HPV genotypes for all cases and unvaccinated cases of CIN2/3, by age group.

		Total	<25 yrs	25-34 yrs	35-44 yrs	45-54 yrs	≥55 yrs
**All cases**							
**N (%)**		3087 (100)	162 (100)	1666 (100)	724 (100)	336 (100)	199 (100)
**HPV genotypes**							
**16**	**Number of infections[1]**	1268.7	48.2	752.2	277.3	129.9	61.2
	**PA % (95% CI)**	41.1 (39.5-42.8)	29.7 (23.0-36.1)	45.1 (42.9-47.5)	38.3 (34.9-41.8)	38.7 (33.7-43.6)	30.7 (24.5-36.9)
	**Cumulative PA %**	41.1 (39.5-42.8)	29.7 (23.0-36.1)	45.1 (42.9-47.5)	38.3 (34.9-41.8)	38.7 (33.7-43.6)	30.7 (24.5-36.9)
**18**	**Number of infections[1]**	206.8	6.0	113.9	57.3	17.7	11.9
	**PA % (95% CI)**	6.7 (5.8-7.6)	3.7 (1.4-6.5)	6.8 (5.6-7.9)	7.9 (5.9-10.1)	5.3 (3.1-7.7)	6.0 (3.0-9.5)
	**Cumulative PA %**	47.8 (46.0-49.5)	33.4 (26.5-39.5)	52.0 (49.6-54.2)	46.2 (42.4-49.7)	43.9 (39.0-49.2)	36.7 (30.7-43.3)
**31/33/45**	**Number of infections[1]**	859.0	46.8	456.1	211.1	94.5	50.5
	**PA % (95% CI)**	27.8 (26.3-29.6)	28.9 (22.1-35.2)	27.4 (25.4-29.5)	29.2 (25.8-32.6)	28.1 (23.4-32.6)	25.4 (19.3-31.4)
	**Cumulative PA %**	75.6 (74.2-77.2)	62.3 (55.4-68.8)	79.4 (77.5-81.2)	75.4 (72.2-78.4)	72.1 (67.1-76.7)	62.1 (55.1-68.6)
**52/58**	**Number of infections[1]**	354.5	22.5	172.7	93.1	38.0	28.3
	**PA % (95% CI)**	11.5 (10.4-12.6)	13.9 (9.2-18.8)	10.4 (9.0-11.9)	12.9 (10.5-15.2)	11.3 (8.0-14.5)	14.2 (9.5-19.3)
	**Cumulative PA %**	87.1 (85.9-88.3)	76.2 (69.8-82.3)	89.7 (88.3-91.2)	88.2 (86.0-90.4)	83.4 (79.5-87.2)	76.3 (70.4-82.1)
**35/39/51 /56/59**	**Number of infections[1]**	250.8	23.7	112.0	57.6	32.8	24.7
	**PA % (95% CI)**	8.1 (7.2-9.1)	14.6 (9.4-20.1)	6.7 (5.5-8.0)	8.0 (6.0-9.9)	9.8 (7.0-12.7)	12.4 (8.1-17.0)
	**Cumulative PA %**	95.2 (94.4-96.0)	90.8 (86.3-94.8)	96.5 (95.6-97.3)	96.2 (94.7-97.4)	93.1 (90.1-95.6)	88.7 (84.4-92.9)
**Probably/ possibly high-risk[3]**	**Number of infections[1]**	121.3	10.8	51.3	22.3	19.7	17.3
	**PA % (95% CI)**	3.9 (3.2-4.7)	6.6 (3.1-10.5)	3.1 (2.3-4.0)	3.1 (2.0-4.4)	5.8 (3.4-8.5)	8.7 (4.9-12.7)
	**Cumulative PA %**	99.2 (98.8-99.5)	97.4 (95.0-99.6)	99.5 (99.2-99.8)	99.3 (98.6-99.8)	99.0 (97.7-99.9)	97.4 (95.2-99.4)
**Low-risk[4]**	**Number of infections[1]**	25.9	4.1	7.8	5.3	3.5	5.1
	**PA % (95% CI)**	0.8 (0.5-1.2)	2.6 (0.4-5.0)	0.5 (0.2-0.8)	0.7 (0.2-1.4)	1.0 (0.1-2.3)	2.6 (0.6-4.8)
	**Cumulative PA %**	100.0	100.0	100.0	100.0	100.0	100.0

		Unvaccinated[2] cases					
		Total	<25 yrs	25-34 yrs	35-44 yrs	45-54 yrs	≥55 yrs
N (%)		2614 (100)	86 (100)	1285 (100)	713 (100)	332 (100)	198 (100)
HPV genotypes							
16	Number of infections[1]	1088.4	31.6	594.1	274.6	127.9	60.2
	PA % (95% CI)	41.6 (39.8-43.6)	36.8 (27.2-45.6)	46.2 (43.8-49.0)	38.5 (35.0-42.0)	38.5 (33.6-43.6)	30.4 (24.2-36.5)
	Cumulative PA % (95% CI)	41.6 (39.8-43.6)	36.8 (27.2-45.6)	46.2 (43.8-49.0)	38.5 (35.0-42.0)	38.5 (33.6-43.6)	30.4 (24.2-36.5)
18	Number of infections[1]	179.1	5.5	86.7	57.3	17.7	11.9
	PA % (95% CI)	6.9 (5.9-7.8)	6.4 (2.3-11.8)	6.7 (5.4-8.0)	8.0 (5.9-10.3)	5.3 (3.2-7.8)	6.0 (3.0-9.5)
	Cumulative PA % (95% CI)	48.5 (46.7-50.4)	43.2 (33.0-52.1)	53.0 (50.4-55.6)	46.5 (42.8-50.1)	43.9 (39.0-49.1)	36.4 (30.3-43.0)
31/33/45	Number of infections[1]	722.7	22.2	349.7	207.8	92.5	50.5
	PA % (95% CI)	27.6 (26.1-29.3)	25.9 (17.2-34.7)	27.2 (25.0-29.8)	29.2 (25.7-32.5)	27.9 (23.0-32.4)	25.5 (19.5-31.5)
	Cumulative PA % (95% CI)	76.1 (74.6-77.9)	69.0 (60.0-77.9)	80.2 (78.1-82.3)	75.7 (72.6-78.7)	71.7 (66.9-76.4)	61.9 (54.9-68.4)
52/58	Number of infections[1]	293.2	7.6	127.3	92.1	38.0	28.3
	PA % (95% CI)	11.2 (10.0-12.4)	8.8 (3.7-14.6)	9.9 (8.3-11.5)	12.9 (10.6-15.3)	11.4 (8.1-14.7)	14.3 (9.5-19.5)
	Cumulative PA % (95% CI)	87.4 (86.1-88.6)	77.8 (69.2-85.6)	90.1 (88.5-91.7)	88.6 (86.4-90.8)	83.2 (79.3-86.9)	76.2 (70.2-82.1)
35/39/51/56/59	Number of infections[1]	213.0	12.6	89.2	53.6	32.8	24.7
	PA % (95% CI)	8.1 (7.1-9.2)	14.7 (8.2-22.0)	6.9 (5.6-8.3)	7.5 (5.7-9.5)	9.9 (7.0-12.9)	12.5 (8.1-17.2)
	Cumulative PA % (95% CI)	95.5 (94.7-96.3)	92.5 (86.9-97.3)	97.0 (96.1-97.9)	96.1 (94.7-97.4)	93.0 (90.0-95.6)	88.7 (84.3-92.9)
Probably/ possibly high-risk[3]	Number of infections[1]	96.0	3.4	33.3	22.3	19.7	17.3
	PA % (95% CI)	3.7 (2.9-4.5)	3.9 (0.4-8.3)	2.6 (1.8-3.5)	3.1 (2.0-4.5)	5.9 (3.4-8.6)	8.8 (4.9-12.8)
	Cumulative PA % (95% CI)	99.2 (98.8-99.5)	96.5 (92.1-100.0)	99.6 (99.3-99.9)	99.3 (98.5-99.8)	98.9 (97.7-99.9)	97.4 (95.1-99.4)
Low-risk[4]	Number of infections[1]	21.6	3.1	4.7	5.3	3.5	5.1
	PA % (95% CI)	0.8 (0.5-1.2)	3.5 (0.05-7.9)	0.4 (0.09-0.7)	0.7 (0.2-1.5)	1.1 (0.10-2.3)	2.6 (0.6-4.9)
	Cumulative PA %	100.0	100.0	100.0	100.0	100.0	100.0

CIN2/3: cervical intraepithelial neoplasia grade 2—3, PA: proportional attribution.

[1] Numbers are not whole due to weighting.

[2]Unvaccinated cases: Not registered with any HPV vaccine dose or received the first dose 365 days or less before they were first diagnosed with CIN2/3.

[3]HPV26/30/53/66/67/68/69/70/73/82

[4]HPV6/11/40/42/43/54/61/74/81/83/86/87/89/90/91

Table S9: Proportional attribution of HPV genotypes in all cases and unvaccinated cases of ICC by age groups.

		Total	<25 yrs	25-34 yrs	35-44 yrs	45-54 yrs	55-64 yrs	65-74 yrs	75+ yrs
**All cases**									
**N (%)**		1265 (100)	12 (100)	248 (100)	346 (100)	241 (100)	188 (100)	145 (100)	85 (100)
**HPV genotypes**									
**16**	**Number of infections[1]**	702.0	5.0	158.6	184.7	117.0	117.7	77.0	42.0
	**PA % (95% CI[2])**	55.5 (52.9-58.3)	41.7 (14.8-70.0)	63.9 (58.3-69.7)	53.4 (48.2-58.7)	48.5 (42.3-54.2)	62.6 (55.7-69.5)	53.1 (44.4-61.4)	49.4 (38.9-60.5)
	**Cumulative PA % (95% CI)**	55.5 (52.9-58.3)	41.7 (14.8-70.0)	63.9 (58.3-69.7)	53.4 (48.2-58.7)	48.5 (42.3-54.2)	62.6 (55.7-69.5)	53.1 (44.4-61.4)	49.4 (38.9-60.5)
**18**	**Number of infections[1]**	241.7	3.0	54.6	88.1	47.7	17.3	22.0	9.0
	**PA % (95% CI[2])**	19.1 (17.0-21.3)	25.0 (0-53.1)	22.0 (17.3-27.4)	25.5 (21.0-29.9)	19.8 (14.8-25.1)	9.2 (5.2-13.7)	15.2 (9.6-21.4)	10.6 (4.5-17.7)
	**Cumulative PA % (95% CI)**	74.6 (72.1-77.1)	66.7 (38.8-91.7)	85.9 (81.4-90.1)	78.8 (74.3-82.9)	68.4 (62.7-74.0)	71.8 (64.9-78.5)	68.3 (60.6-76.2)	60.0 (49.7-70.5)
**31/33/ 45**	**Number of infections[1]**	193.8	3.0	29.6	52.1	46.2	27.0	21.0	14.9
	**PA % (95% CI[2])**	15.3 (13.4-17.2)	25.0 (0-55.1)	11.9 (8.2-16.1)	15.1 (11.6-18.8)	19.2 (14.6-24.3)	14.4 (9.5-19.6)	14.5 (8.6-20.5)	17.6 (9.6-26.5)
	**Cumulative PA % (95% CI)**	89.9 (88.1-91.6)	91.7 (75.0-100.0)	97.9 (96.0-99.5)	93.9 (90.9-96.3)	87.5 (83.7-91.5)	86.2 (80.9-90.9)	82.8 (76.4-88.6)	77.6 (68.3-86.1)
**52/58**	**Number of infections[1]**	32.0	0	0.3	6.0	6.9	7.7	6.0	5.0
	**PA % (95% CI[2])**	2.5 (1.7-3.5)	0 (NA)	0.1 (0-0.4)	1.7 (0.6-3.3)	2.9 (0.8-5.0)	4.1 (1.5-7.1)	4.1 (1.3-7.6)	5.9 (1.2-11.6)
	**Cumulative PA % (95% CI)**	92.4 (90.9-93.8)	91.7 (75.0-100.0)	98.0 (96.2-99.6)	95.6 (93.1-97.6)	90.4 (86.6-93.8)	90.3 (85.4-94.1)	86.9 (81.4-91.9)	83.5 (75.5-90.8)
**35/39/ 51/56/ 59**	**Number of infections[1]**	50.6	1.0	2.1	8.1	10.3	10.3	9.9	9.0
	**PA % (95% CI[2])**	4.0 (2.9-5.2)	8.3 (0-25.0)	0.8 (0.008-2.2)	2.3 (0.9-4.1)	4.3 (1.9-6.8)	5.5 (2.6-8.9)	6.9 (3.3-11.4)	10.6 (5.1-17.7)
	**Cumulative PA % (95% CI)**	96.4 (95.4-97.5)	100.0 (NA)	98.8 (97.4-100.0)	98.0 (96.4-99.2)	94.7 (91.8-97.2)	95.7 (92.3-98.4)	93.7 (89.6-97.6)	94.0 (88.8-98.7)
**Probably /possibly high-risk[3]**	**Number of infections**	39.9	0	2.9	7.1	12.8	6.0	7.1	4.0
	**PA % (95% CI[2])**	3.2 (2.2-4.2)	0 (NA)	1.2 (0.01-2.6)	2.0 (0.8-3.6)	5.3 (2.8-8.2)	3.2 (1.0-6.2)	4.9 (1.4-8.7)	4.7 (1.0-9.5)
	**Cumulative PA % (95% CI)**	99.6 (99.2-99.9)	100.0 (NA)	99.998 (99.989-100.0)	99.998 (99.992-100.0)	99.998 (99.991-100.0)	98.9 (97.3-100.0)	98.6 (96.4-100.0)	98.8 (96.3-100.0)
**Low-risk[4]**	**Number of infections[1]**	5.1	0	0.004	0.006	0.004	2.0	2.0	1.1
	**PA % (95% CI[2])**	0.4 (0.08-0.8)	0 (NA)	0.002 (0-0.01)	0.002 (0-0.008)	0.002 (0-0.009)	1.1 (0-2.7)	1.4 (0-3.6)	1.2 (0-3.7)
	**Cumulative PA %**	100.0	100.0	100.0	100.0	100.0	100.0	100.0	100.0

		Total	<25 yrs	25-34 yrs	35-44 yrs	45-54 yrs	55-64 yrs	65-74 yrs	75+ yrs
					Unvaccinated cases[5]				
N (%)		1221 (100)	6.0 (100)	211 (100)	345 (100)	241 (100)	188 (100)	145 (100)	85 (100)
HPV genotypes									
16	Number of infections[1]	669.0	1.0	129.6	184.7	117.0	117.7	77.0	42.0
	PA % (95% CI[2])	54.8 (52.1-57.7)	16.7 (0-50.0)	61.4 (54.9-67.9)	53.5 (48.3-59.1)	48.5 (42.3-54.3)	62.6 (55.7-69.6)	53.1 (44.4-61.5)	49.4 (38.8-60.5)
	Cumulative PA % (95% CI)	54.8 (52.1-57.7)	16.7 (0-50.0)	61.4 (54.9-67.9)	53.5 (48.3-59.1)	48.5 (42.3-54.3)	62.6 (55.7-69.6)	53.1 (44.4-61.5)	49.4 (38.8-60.5)
18	Number of infections[1]	232.7	1.0	48.6	87.1	47.7	17.3	22.0	9.0
	PA % (95% CI[2])	19.1 (16.9-21.3)	16.7 (0-50.0)	23.0 (17.4-28.9)	25.2 (20.8-29.7)	19.8 (14.8-25.1)	9.2 (5.2-13.7)	15.2 (9.6-21.4)	10.6 (4.5-17.7)
	Cumulative PA % (95% CI)	73.8 (71.3-76.3)	33.3 (0.0-80.0)	84.4 (79.3-89.2)	78.8 (74.3-82.9)	68.4 (62.6-74.0)	71.8 (64.9-78.5)	68.3 (60.6-76.3)	60.0 (49.5-70.5)
31/33/45	Number of infections[1]	192.8	3.0	28.6	52.1	46.2	27.0	21.0	14.9
	PA % (95% CI[2])	15.8 (13.8-17.8)	50.0 (0-100.0)	13.5 (9.3-18.3)	15.1 (11.6-18.8)	19.2 (14.6-24.3)	14.4 (9.5-19.6)	14.5 (8.5-20.6)	17.6 (9.6-26.5)
	Cumulative PA % (95% CI)	89.6 (87.8-91.3)	83.3 (50.0-100.0)	98.0 (95.9-99.7)	93.9 (90.9-96.3)	87.5 (83.7-91.5)	86.2 (80.9-90.9)	82.8 (76.4-88.7)	77.6 (68.2-86.1)
52/58	Number of infections[1]	32.0	0	0.3	6.0	6.9	7.7	6.0	5.0
	PA % (95% CI[2])	2.6 (1.8-3.6)	0 (NA)	0.2 (0-0.5)	1.7 (0.6-3.3)	2.9 (0.8-5.0)	4.1 (1.5-7.1)	4.1 (1.3-7.6)	5.9 (1.2-11.6)
	Cumulative PA % (95% CI)	92.3 (90.6-93.7)	83.3 (50.0-100.0)	98.1 (96.1-99.7)	95.6 (93.0-97.6)	90.4 (86.6-93.8)	90.3 (85.4-94.1)	86.9 (81.4-92.0)	83.5 (75.4-90.9)
35/39/51/56/59	Number of infections[1]	49.6	1.0	1.1	8.1	10.3	10.3	9.9	9.0
	PA % (95% CI[2])	4.1 (3.0-5.3)	16.7 (0-50.0)	0.5 (0-1.7)	2.3 (0.9-4.1)	4.3 (1.8-6.8)	5.5 (2.6-8.9)	6.9 (3.3-11.4)	10.6 (5.1-17.7)
	Cumulative PA % (95% CI)	96.3 (95.2-97.4)	100.0 (NA)	98.6 (97.0-100.0)	97.9 (96.3-99.1)	94.7 (91.8-97.2)	95.7 (92.3-98.4)	93.7 (89.6-97.6)	94.0 (88.8-98.7)
Probably /possibly high-risk[3]	Number of infections[1]	39.9	0	2.9	7.1	12.8	6.0	7.1	4.0
	PA % (95% CI[2])	3.3 (2.2-4.3)	0 (NA)	1.4 (0.02-3.0)	2.1 (0.8-3.7)	5.3 (2.8-8.2)	3.2 (1.0-6.2)	4.9 (1.4-8.7)	4.7 (1.0-9.5)
	Cumulative PA % (95% CI)	99.6 (99.2-99.9)	100.0 (NA)	99.998 (99.988-100.0)	99.998 (99.992-100.0)	99.998 (99.991-100.0)	98.9 (97.3-100.0)	98.6 (96.4-100.0)	98.8 (96.3-100.0)
Low-risk[4]	Number of infections[1]	5.1	0	0.004	0.006	0.004	2.0	2.0	1.1
	PA % (95% CI[2])	0.4 (0.08-0.8)	0 (NA)	0.002 (0-0.01)	0.002 (0-0.008)	0.002 (0-0.009)	1.1 (0-2.7)	1.4 (0-3.6)	1.2 (0-3.7)
	Cumulative PA %	100.0	100.0	100.00	100.0	100.0	100.0	100.0	100.0

ICC: Invasive cervical cancer

[1]Numbers are not whole due to weighting

[2]95% CI not calculated if the number of weighted cases is 0.

[3]HPV26/30/53/66/67/68/69/70/73/82

[4]HPV6/11/40/42/43/54/61/74/81/83/86/87/89/90/91

[5] Unvaccinated cases: Not registered with any HPV vaccine doses or the first dose received 365 days or less before they were first diagnosed with cervical intraepithelial neoplasia grade 2—3, adenocarcinoma in situ or ICC.

Table S10: Morphology of control sections[1] of all genotyped samples from cases reported during 2017—2021.

	CIN2/3 N = 3237		AIS N = 714		ICC N = 1362	
	Confirmed[2] cases N = 3087 (95.4%)	Unconfirmed[3] cases N = 150 (4.6%)	Confirmed[2] cases N = 550 (77.0%)	Unconfirmed[3] cases N = 164 (23.0%)	Confirmed[2] cases N = 1265 (92.9%)	Unconfirmed[3] cases N = 97 (7.1%)
**Control sections[1] combinations**	**n (%)**	**n (%)**	**n (%)**	**n (%)**	**n (%)**	**n (%)**
CIN1 & CIN1	0 (0)	65 (43.3)	0 (0)	20 (12.2)	0 (0)	2 (2.1)
CIN2/3 & CIN2/3	2858 (92.6)	0 (0)	0 (0)	101 (61.6)	0 (0)	73 (75.3)
AIS & AIS	0 (0)	4 (2.7)	506 (92.0)	0 (0)	0 (0)	12 (12.4)
ICC & ICC	0 (0)	20 (13.3)	0 (0)	12 (7.3)	1202 (95.0)	0 (0)
None/NA & None/NA	0 (0)	44 (29.3)	0 (0)	18 (11.0)	0 (0)	4 (4.1)
CIN1 & CIN2/3	164 (5.3)	0 (0)	0 (0)	9 (5.5)	0 (0)	2 (2.1)
CIN1 & AIS	0 (0)	0 (0)	7 (1.3)	0 (0)	0 (0)	0 (0)
CIN1 & None/NA	0 (0)	16 (10.7)	0 (0)	2 (1.2)	0 (0)	1 (1.0)
CIN2/3 & AIS	4 (0.1)	0 (0)	22 (4.0)	0 (0)	0 (0)	1 (1.0)
CIN2/3 & ICC	7 (0.2)	0 (0)	0 (0)	1 (0.6)	51 (4.0)	0 (0)
CIN2/3 & None/NA	54 (1.7)	0 (0)	0 (0)	1 (0.6)	0 (0)	2 (2.1)
AIS & ICC	0 (0)	0 (0)	1 (0.2)	0 (0)	9 (0.7)	0 (0)
AIS & None/NA	0 (0)	1 (0.7)	14 (2.5)	0 (0)	0 (0)	0 (0)
ICC & None/NA	0 (0)	0 (0)	0 (0)	0 (0)	3 (0.2)	0 (0)

CIN2/3: Cervical intraepithelial neoplasia grade 2 or 3, AIS: Adenocarcinoma in situ, ICC: Invasive cervical cancer, CIN1: Cervical intraepithelial neoplasia grade 1, None/NA: No HPV - induced cervical lesions observed in the control section or the control section was not reviewed.

[1]Control sections: One haematoxylin-eosin-stained section before and after the HPV genotyped section.

[2]Confirmed cases: The histopathological diagnosis was confirmed in at least one control section.

[3]Unconfirmed cases: The histopathological diagnosis not confirmed in either control sections.

Table S11: Crude prevalence of HPV genotypes in cases reported during 2017—2021, with histopathologically confirmed control section(s) versus unconfirmed cases.

	CIN2/3 N = 3 237			AIS N = 714			ICC N = 1 362		
	Confirmed[1] cases N= 3087	Unconfirmed[2] cases N = 150		Confirmed[1] cases N = 550	Unconfirmed[2] cases N = 164		Confirmed[1] cases N = 1 265	Unconfirmed[2] cases N = 97	
	n (%)	n (%)	Mid-p	n (%)	n (%)	Mid-p	n (%)	n (%)	Mid-p
**High-risk HPV types**									
16	1303 (42.2)	46 (30.7)	0.004	224 (40.7)	54 (32.9)	0.08	704 (55.7)	59 (60.8)	0.32
18	270 (8.7)	13 (8.7)	0.94	321 (58.4)	54 (32.9)	<0.001	248 (19.6)	17 (17.5)	0.64
31	558 (18.1)	18 (12.0)	0.06	32 (5.8)	22 (13.4)	0.002	53 (4.2)	3 (3.1)	0.69
33	304 (9.8)	14 (9.3)	0.94	7 (1.3)	4 (2.4)	0.21	67 (5.3)	4 (4.1)	0.72
35	95 (3.1)	4 (2.7)	0.90	3 (0.5)	1 (0.6)	0.79	10 (0.8)	2 (2.1)	0.13
39	79 (2.6)	7 (4.7)	0.09	6 (1.1)	4 (2.4)	0.19	16 (1.3)	0 (0)	0.47
45	132 (4.3)	4 (2.7)	0.47	37 (6.7)	9 (5.5)	0.65	81 (6.4)	7 (7.2)	0.59
51	123 (4.0)	2 (1.3)	0.10	5 (0.9)	5 (3.0)	0.03	3 (0.2)	1 (1.0)	0.14
52	299 (9.7)	8 (5.3)	0.07	11 (2.0)	7 (4.3)	0.12	25 (2.0)	3 (3.1)	0.35
56	57 (1.8)	7 (4.7)	0.02	4 (0.7)	2 (1.2)	0.49	15 (1.2)	2 (2.1)	0.23
58	150 (4.9)	6 (4.0)	0.77	3 (0.5)	4 (2.4)	0.03	10 (0.8)	1 (1.0)	0.37
59	41 (1.3)	4 (2.7)	0.10	5 (0.9)	1 (0.6)	0.81	13 (1.0)	1 (1.0)	0.81
**Probably/Possibly high-risk HPV types**									
26	6 (0.2)	1 (0.7)	-	0 (0)	1 (0.6)	-	0 (0)	0 (0)	-
30	16 (0.5)	1 (0.7)	0.37	0 (0)	2 (1.2)	-	3 (0.2)	0 (0)	-
53	38 (1.2)	4 (2.7)	0.09	1 (0.2)	3 (1.8)	-	1 (0.1)	1 (1.0)	-
66	39 (1.3)	6 (4.0)	0.01	2 (0.4)	4 (2.4)	-	1 (0.1)	0 (0)	-
67	29 (0.9)	2 (1.3)	0.53	2 (0.4)	3 (1.8)	-	2 (0.2)	0 (0)	-
68	33 (1.1)	3 (2.0)	0.16	2 (0.4)	3 (1.8)	-	10 (0.8)	0 (0)	0.76
69	4 (0.1)	0 (0)	-	0 (0)	0 (0)	-	0 (0)	0 (0)	-
70	27 (0.9)	3 (2.0)	0.10	5 (0.9)	0 (0)	-	7 (0.6)	1 (1.0)	-
73	11 (0.4)	2 (1.3)	0.07	0 (0)	0 (0)	-	20 (1.6)	0 (0)	0.28
82	56 (1.8)	4 (2.7)	0.28	0 (0)	1 (0.6)	-	11 (0.9)	1 (1.0)	0.40

CIN2/3 N = 3 237				AIS N = 714			ICC N = 1 362		
	Confirmed[1] cases N= 3087	Unconfirmed[2] cases N = 150		Confirmed[1] cases N = 550	Unconfirmed[2] cases N = 164		Confirmed[1] cases N = 1 265	Unconfirmed[2] cases N = 97	
	n (%)	n (%)	Mid-p	n (%)	n (%)	Mid-p	n (%)	n (%)	Mid-p
**Low-risk HPV types**									
6	28 (0.9)	3 (2.0)	0.11	3 (0.5)	2 (1.2)	-	4 (0.3)	0 (0)	-
11	11 (0.4)	0 (0)	0.70	1 (0.2)	1 (0.6)	-	0 (0)	0 (0)	-
40	4 (0.1)	1 (0.7)	-	0 (0)	0 (0)	-	2 (0.2)	0 (0)	-
42	16 (0.5)	1 (0.7)	0.37	1 (0.2)	1 (0.6)	-	4 (0.3)	0 (0)	-
43	9 (0.3)	2 (1.3)	0.05	0 (0)	0 (0)	-	1 (0.1)	0 (0)	-
54	4 (0.1)	0 (0)	-	0 (0)	1 (0.6)	-	0 (0)	0 (0)	-
61	3 (0.1)	0 (0)	-	1 (0.2)	0 (0)	-	0 (0)	0 (0)	-
74	10 (0.3)	2 (1.3)	0.06	0 (0)	0 (0)	-	0 (0)	0 (0)	-
81	6 (0.2)	0 (0)	-	2 (0.4)	0 (0)	-	0 (0)	0 (0)	-
83	10 (0.3)	1 (0.7)	0.25	0 (0)	0 (0)	-	0 (0)	0 (0)	-
86	1 (0.0)	0 (0)	-	1 (0.2)	0 (0)	-	0 (0)	0 (0)	-
87	6 (0.2)	0 (0)	-	0 (0)	1 (0.6)	-	1 (0.1)	0 (0)	-
89	3 (0.1)	1 (0.7)	-	0 (0)	0 (0)	-	0 (0)	0 (0)	-
90	5 (0.2)	2 (1.3)	-	0 (0)	0 (0)	-	0 (0)	0 (0)	-
91	19 (0.6)	3 (2.0)	0.048	0 (0)	1 (0.6)	-	2 (0.2)	0 (0)	-

CIN2/3: cervical intraepithelial neoplasia grade 2—3, AIS: adenocarcinoma in situ, ICC: invasive cervical cancer, mid-p: Crude prevalences in confirmed and unconfirmed cases were compared using Fisher's exact test and corresponding mid-p-values. A mid-p-value less than 0.05 was considered statistically significant. The test was performed for all high-risk human papillomavirus types and for other types where the total number of genotype-specific positive cases across the two groups was more than 10.

[1]Confirmed cases: The histopathological diagnosis was confirmed in at least one haematoxylin-eosin-stained section before or after the HPV genotyped section.

[2]Unconfirmed cases: The histopathological diagnosis was not confirmed in either haematoxylin-eosin-stained section before or after the HPV genotyped section.

Table S12: Proportional attribution of HPV genotypes in cases reported during 2017—2021, including specimens where histopathological diagnosis is not confirmed in either control sections[1].

	CIN2/3<br>*N = 3237*	*AIS*<br>*N = 714*	*ICC*<br>*N = 1362*
	**n[2] (%)**	**n[2] (%)**	**n[2] (%)**
**High-risk HPV types**			
16	1313.6 (40.6)	249.7 (35.0)	760.8 (55.9)
18	219.5 (6.8)	353.2 (49.5)	258.6 (19.0)
31	504.2 (15.6)	24.5 (3.4)	51.5 (3.8)
33	277.4 (8.6)	3.7 (0.5)	71.0 (5.2)
35	63.9 (2.0)	1.0 (0.1)	10.6 (0.8)
39	59.3 (1.8)	3.1 (0.4)	15.0 (1.1)
45	110.5 (3.4)	42.5 (5.9)	83.4 (6.1)
51	79.9 (2.5)	5.1 (0.7)	1.0 (0.1)
52	242.1 (7.5)	4.3 (0.6)	27.7 (2.0)
56	39.1 (1.2)	2.0 (0.3)	16.9 (1.2)
58	124.6 (3.8)	2.7 (0.4)	8.4 (0.6)
59	27.1 (0.8)	2.4 (0.3)	11.2 (0.8)
**Probably/possibly high-risk HPV types**			
26	3.3 (0.1)	1.0 (0.1)	0 (0)
30	5.0 (0.2)	1.3 (0.2)	2.1 (0.2)
53	21.9 (0.7)	1.0 (0.1)	0 (0)
66	25.1 (0.8)	1.3 (0.2)	1.0 (0.1)
67	12.8 (0.4)	2.1 (0.3)	2.0 (0.2)
68	15.5 (0.5)	3.0 (0.4)	9.0 (0.7)
69	1.0 (0)	0 (0)	0 (0)
70	15.8 (0.5)	3.0 (0.4)	6.1 (0.4)
73	7.1 (0.2)	0 (0)	18.5 (1.4)
82	32.5 (1.0)	1.0 (0.1)	2.2 (0.2)
**Low-risk HPV types**			
6	12.5 (0.4)	2.0 (0.3)	1.0 (0.1)
11	3.1 (0.1)	0 (0)	0 (0)
40	0 (0)	0 (0)	0 (0)
42	4.9 (0.2)	1.0 (0.1)	3.1 (0.2)
43	1.2 (0.0)	0 (0)	0 (0)
54	0 (0)	0 (0)	0 (0)
61	1.0 (0)	1.0 (0.1)	0 (0)
74	1.5 (0.1)	0 (0)	0 (0)
81	3.2 (0.1)	1.0 (0.1)	0 (0)
83	3.3 (0.1)	0 (0)	0 (0)
86	0 (0)	0 (0)	0 (0)
87	0 (0)	0 (0)	0 (0)
89	1.0 (0)	0 (0)	0 (0)

	CIN2/3<br>*N = 3237*	*AIS*<br>*N = 714*	*ICC*<br>*N = 1362*
	**n[2] (%)**	**n[2] (%)**	**n[2] (%)**
90	1.0 (0)	0 (0)	0 (0)
91	3.0 (0.1)	1.0 (0.1)	1.0 (0.1)

CIN2/3: cervical intraepithelial neoplasia grade 2 or 3, AIS: adenocarcinoma in situ, ICC: invasive cervical cancer.
[1]Control section: One haematoxylin-eosin-stained section before and after the HPV genotyped section.
[2]Numbers are not whole due to weighting.

Table S13: Human papillomavirus (HPV) vaccination status in vaccine-eligible cases (born 1991 or later) reported during 2017—2024, by morphology and birth cohort.

	Unvaccinated[1]	Vaccinated 1 dose[2]	Vaccinated 2 doses[3]	Vaccinated ≥3 doses[4]	Total
**CIN2/3**	**n (%)**	**n (%)**	**n (%)**	**n (%)**	**n (%)**
Catch-up-eligible cases[5]	668 (43.3)	75 (4.9)	152 (9.9)	646 (41.9)	1541 (100.0)
Routine-eligible cases[6]	35 (22.7)	NA*	8 (5.2)	111 (72.1)	154 (100.0)
**AIS**					
Catch-up-eligible cases[5]	157 (35.3)	26 (5.8)	53 (11.9)	209 (47.0)	445 (100.0)
Routine-eligible cases[6]	13 (52.0)	NA**	NA**	12 (48.0)	25 (100.0)
**ICC**					
Catch-up-eligible cases[5]	62 (45.9)	8 (5.9)	15 (11.1)	50 (37.0)	135 (100.0)
Routine-eligible cases[6]	0 (0)	0 (0)	0 (0)	5 (100.0)	5 (100.0)

CIN2/3: cervical intraepithelial neoplasia grade 2—3, AIS: adenocarcinoma in situ, ICC: invasive cervical cancer.

[1]Unvaccinated: not registered with any HPV vaccine doses or the first dose received 365 days or less before they were first diagnosed with CIN2/3, AIS or ICC.

[2]Vaccinated 1 dose: received one dose of HPV vaccine more than 365 days before they were first diagnosed with CIN2/3, AIS or ICC.

[3]Vaccinated 2 doses: received two doses of HPV vaccine more than 365 days before they were first diagnosed with CIN2/3, AIS or ICC.

[4]Vaccinated ≥3 doses: received at least three doses of HPV vaccine more than 365 days before they were first diagnosed with CIN2/3, AIS or ICC.

[5]Catch-up eligible cases: born 1991—1996.

[6]Routine-eligible cases: born 1997 or later.

*Since there were fewer than 5 cases vaccinated with 1 dose, they were anonymized by reporting them together with cases vaccinated with 2 doses.

** Since there were fewer than 5 cases vaccinated with 1 dose and 2 doses, they were anonymized by reporting them together with cases vaccinated with 3 doses.

Table S14: Proportional attribution of HPV genotypes in catch-up-eligible cases (born 1991—1996) reported during 2017—2024[1], by morphology and vaccination status.

	CIN2/3				AIS				ICC			
	Unvaccinated[2] N = 668		Vaccinated[3] N = 873		Unvaccinated[2] N = 157		Vaccinated[3] N = 288		Unvaccinated[2] N = 62		Vaccinated[3] N = 73	
	n	PA % (95% CI)	n	PA % (95% CI)	n	% (95% CI)	n	% (95% CI)	n	% (95% CI)	n	% (95% CI)
**16**	305.0	45.7 (41.9-49.7)	309.7	35.5 (32.5-38.6)	60.2	38.3 (31.2-45.2)	109.4	38.0 (32.6-43.4)	38.7	62.5 (50.3-73.4)	46.0	63.0 (52.2-74.0)
**18**	37.6	5.6 (4.1-7.3)	50.8	5.8 (4.5-7.3)	88.0	56.0 (48.7-63.3)	156.1	54.2 (48.2-59.9)	9.8	15.8 (7.6-25.7)	14.0	19.2 (10.8-28.6)
**31**	101.3	15.2 (12.6-17.9)	163.8	18.8 (16.5-21.2)	1.3	0.8 (0.10-2.2)	1.3	0.5 (0.07-1.2)	1.2	1.9 (0-5.6)	1.0	1.4 (0-4.4)
**33**	63.2	9.5 (7.4-11.5)	90.2	10.3 (8.4-12.3)	0	0 (NA)	1.0	0.3 (0-0)	5.0	8.1 (1.7-15.5)	6.0	8.2 (2.7-14.8)
**35**	11.6	1.7 (0.9-2.6)	14.4	1.7 (0.9-2.5)	0	0 (NA)	0	0 (NA)	1.0	1.6 (0-5.4)	0	0 (NA)
**39**	13.4	2.0 (1.1-3.1)	22.1	2.5 (1.6-3.6)	0.004	0.003 (0-0.009)	1.0	0.4 (0.001-1.1)	0.04	0.07 (0-0.2)	2.0	2.7 (0-7.1)
**45**	15.3	2.3 (1.3-3.5)	20.7	2.4 (1.5-3.4)	4.5	2.9 (0.7-5.6)	11.2	3.9 (1.8-6.2)	4.2	6.8 (1.6-13.6)	2.0	2.7 (0-6.8)
**51**	20.1	3.0 (1.9-4.3)	25.2	2.9 (1.9-4.0)	0	0 (NA)	0	0 (NA)	0	0 (NA)	0	0 (NA)
**52**	42.1	6.3 (4.7-8.0)	80.3	9.2 (7.4-11.1)	0	0 (NA)	1.0	0.3 (0-0)	0	0 (NA)	0	0 (NA)
**56**	2.7	0.4 (0.06-0.9)	5.1	0.6 (0.2-1.1)	0.01	0.007 (0-0.02)	0.01	0.004 (0-0.010)	0	0 (NA)	1.0	1.4 (0-4.6)
**58**	23.3	3.5 (2.3-4.7)	31.0	3.5 (2.4-4.6)	0	0 (NA)	0	0 (NA)	0.3	0.5 (0-1.5)	0	0 (NA)
**59**	4.5	0.7 (0.2-1.3)	7.4	0.9 (0.4-1.4)	0.006	0.004 (0-0.01)	4.0	1.4 (0.3-2.9)	1.0	1.6 (0-5.6)	0	0 (NA)
**26**	3.0	0.4	1.0	0.1	0	0	0	0	0	0	1.0	1.4

	CIN2/3				AIS				ICC			
	Unvaccinated[2] N = 668		Vaccinated[3] N = 873		Unvaccinated[2] N = 157		Vaccinated[3] N = 288		Unvaccinated[2] N = 62		Vaccinated[3] N = 73	
	**n**	**PA % (95% CI)**	**n**	**PA % (95% CI)**	**n**	**% (95% CI)**	**n**	**% (95% CI)**	**n**	**% (95% CI)**	**n**	**% (95% CI)**
		(0.0008-0.9)		(0.001-0.4)		(NA)		(NA)		(NA)		(0-0)
**30**	1.5	0.2 (0.02-0.6)	2.4	0.3 (0.01-0.6)	0	0 (NA)	0	0 (NA)	0	0 (NA)	0	0 (NA)
**53**	1.3	0.2 (0.006-0.5)	2.3	0.3 (0.03-0.6)	0	0 (NA)	0	0 (NA)	0	0 (NA)	0	0 (NA)
**66**	3.9	0.6 (0.1-1.1)	7.5	0.9 (0.3-1.6)	0	0 (NA)	1.0	0.3 (0-1.1)	0	0 (NA)	0	0 (NA)
**67**	1.4	0.2 (0.004-0.6)	8.1	0.9 (0.4-1.6)	0	0 (NA)	0	0 (NA)	0	0 (NA)	0	0 (NA)
**68**	0.1	0.02 (0-0.04)	6.1	0.7 (0.3-1.3)	0	0 (NA)	1.0	0.3 (0-1.1)	0	0 (NA)	0	0 (NA)
**69**	0.0009	0.0001 (0-0.0004)	0	0 (NA)	0	0 (NA)	0	0 (NA)	0	0 (NA)	0	0 (NA)
**70**	1.2	0.2 (0.002-0.5)	1.3	0.2 (0.01-0.4)	2.0	1.3 (0-3.3)	1.0	0.3 (0-1.1)	0	0 (NA)	0	0 (NA)
**73**	2.0	0.3 (0.0006-0.8)	3.3	0.4 (0.03-0.8)	0	0 (NA)	0	0 (NA)	0.7	1.1 (0-3.6)	0	0 (NA)
**82**	4.1	0.6 (0.1-1.2)	13.8	1.6 (0.8-2.4)	0	0 (NA)	0	0 (NA)	0	0 (NA)	0	0 (NA)
**6**	4.2	0.6 (0.1-1.2)	3.4	0.4 (0.08-0.8)	1.0	0.6 (0-2.2)	0	0 (NA)	0	0 (NA)	0	0 (NA)
**11**	2.0	0.3 (0.001-0.8)	0.1	0.01 (0.0009-0.03)	0	0 (NA)	0	0 (NA)	0	0 (NA)	0	0 (NA)
**40**	0	0 (NA)	0	0 (NA)	0	0 (NA)	0	0 (NA)	0	0 (NA)	0	0 (NA)
**42**	1.1	0.2	0.7	0.09	0	0	0	0	0	0	0	0

	CIN2/3				AIS				ICC			
	Unvaccinated[2] N = 668		Vaccinated[3] N = 873		Unvaccinated[2] N = 157		Vaccinated[3] N = 288		Unvaccinated[2] N = 62		Vaccinated[3] N = 73	
	n	PA % (95% CI)	n	PA % (95% CI)	n	% (95% CI)	n	% (95% CI)	n	% (95% CI)	n	% (95% CI)
		(0-0.5)		(0.01-0.2)		(NA)		(NA)		(NA)		(NA)
**43**	0	0 (NA)	0	0 (NA)	0	0 (NA)	0	0 (NA)	0	0 (NA)	0	0 (NA)
**54**	0	0 (NA)	0	0 (NA)	0	0 (NA)	0	0 (NA)	0	0 (NA)	0	0 (NA)
**61**	0	0 (NA)	0.002	0.0002 (0-0.0005)	0	0 (NA)	0	0 (NA)	0	0 (NA)	0	0 (NA)
**74**	0	0 (NA)	1.0	0.1 (0-0)	0	0 (NA)	0	0 (NA)	0	0 (NA)	0	0 (NA)
**81**	1.0	0.2 (0-0.5)	0.2	0.02 (0-0.06)	0.004	0.003 (0-0.009)	0.01	0.004 (0-0.010)	0	0 (NA)	0	0 (NA)
**83**	0	0 (NA)	0.04	0.004 (0-0.01)	0	0 (NA)	0	0 (NA)	0	0 (NA)	0	0 (NA)
**86**	0	0 (NA)	0	0 (NA)	0	0 (NA)	0	0 (NA)	0	0 (NA)	0	0 (NA)
**87**	0	0 (NA)	1.0	0.1 (0-0)	0	0 (NA)	0	0 (NA)	0	0 (NA)	0	0 (NA)
**89**	0	0 (NA)	0	0 (NA)	0	0 (NA)	0	0 (NA)	0	0 (NA)	0	0 (NA)
**90**	0	0 (NA)	0	0 (NA)	0	0 (NA)	0	0 (NA)	0	0 (NA)	0	0 (NA)
**91**	1.0	0.2 (0.0001-0.5)	0.02	0.003 (0.0004-0.006)	0	0 (NA)	0	0 (NA)	0	0 (NA)	0	0 (NA)

CIN2/3: cervical intraepithelial neoplasia grade 2 or 3, AIS: adenocarcinoma in situ, ICC: invasive cervical cancer, PA: proportional attribution, CI: confidence interval.
[1]Genotyping of cases reported during 2024 not complete at the time of data retrieval.
[2]Unvaccinated: not registered with any HPV vaccine doses or the first dose received 365 days or less before they were first diagnosed with CIN2/3, AIS or ICC.

[3]Vaccinated: received at least one dose of HPV vaccine more than 365 days before they were first diagnosed with CIN2/3, AIS or ICC.

Table S15: Proportional attribution of HPV genotypes by vaccination status in routine-eligible cases (born 1997 or later) with cervical intraepithelial neoplasia grade 2—3 (CIN2/3), reported during 2017—2024[1].

HPV type	Unvaccinated[2] N = 35		Vaccinated before age 15 years[3] N = 95		Vaccinated at age 15 years or later[4] N = 24	
	n[5]	PA % (95% CI)	n[5]	PA % (95% CI)	n[5]	PA % (95% CI)
**High risk HPV types**						
**16**	17.5	49.9 (33.7-66.1)	1.0	1.1 (0-3.4)	7.0	29.2 (12.5-48.1)
**18**	1.5	4.2 (0-11.1)	0	0 (NA)	1.0	4.2 (0-12.8)
**31**	1.3	3.6 (0-10.2)	5.5	5.8 (1.9-10.5)	1.0	4.2 (0-13.6)
**33**	1.2	3.3 (0-10.2)	16.7	17.6 (10.6-25.7)	1.8	7.6 (0-19.4)
**35**	1.0	2.9 (0-9.3)	5.0	5.3 (1.1-9.6)	1.9	7.8 (0-19.1)
**39**	0.08	0.2 (0-0.6)	4.4	4.6 (1.2-8.7)	1.2	4.8 (0-12.5)
**45**	1.0	2.9 (0-9.5)	4.9	5.1 (1.0-10.1)	1.0	4.2 (0-13.6)
**51**	2.0	5.7 (0-14.7)	8.6	9.0 (4.2-14.3)	1.2	5.2 (0-14.1)
**52**	5.1	14.5 (5.1-26.9)	16.7	17.6 (10.8-24.8)	2.8	11.5 (0-25.1)
**56**	0	0 (NA)	3.0	3.2 (0-7.1)	1.1	4.7 (0-14.9)
**58**	1.4	3.9 (0-10.6)	17.7	18.6 (11.2-26.6)	1.7	6.9 (0-17.4)
**59**	0.02	0.05 (0-0.2)	1.5	1.5 (0.09-3.9)	0	0 (NA)
**Probably/possibly high-risk HPV types**						
**26**	1.0	2.9 (0-9.0)	0	0 (NA)	0.2	0.8 (0-3.0)
**30**	0.01	0.04 (0-0.1)	1.2	1.3 (0-4.0)	0.02	0.07 (0-0.2)
**53**	0	0 (NA)	1.1	1.2 (0-3.0)	1.0	4.2 (0-13.7)
**66**	0.08	0.2 (0-0.8)	1.2	1.3 (0-3.7)	0	0 (NA)
**67**	0	0 (NA)	0.7	0.8 (0-2.6)	0.04	0.2 (0-0.6)
**68**	0	0 (NA)	0.4	0.4 (0-1.1)	0	0 (NA)
**69**	0	0 (NA)	0	0 (NA)	0	0 (NA)
**70**	0.01	0.03 (0-0.10)	1.0	1.1 (0-3.4)	0	0 (NA)
**73**	1.0	2.9 (0-9.1)	1.3	1.4 (0-3.9)	0	0 (NA)
**82**	0	0 (NA)	3.0	3.2 (0-6.7)	0.3	1.3 (0-4.5)
**Low-risk HPV types**						
**6**	0.007	0.02 (0-0.07)	0	0 (NA)	0.8	3.3 (0-12.2)
**11**	0	0 (NA)	0	0 (NA)	0	0 (NA)
**40**	0	0 (NA)	0	0 (NA)	0	0 (NA)
**42**	0	0 (NA)	0.04	0.04 (0-0.1)	0	0 (NA)
**43**	0	0 (NA)	0	0 (NA)	0	0 (NA)
**54**	0	0 (NA)	0	0 (NA)	0	0 (NA)
**61**	0	0 (NA)	0	0 (NA)	0	0 (NA)
**74**	0	0 (NA)	0	0 (NA)	0	0 (NA)
**81**	1.0	2.9 (0-9.0)	0	0 (NA)	0	0 (NA)
**83**	0	0 (NA)	0.03	0.04 (0-0.1)	0	0 (NA)
**86**	0	0 (NA)	0	0 (NA)	0	0 (NA)
**87**	0	0 (NA)	0	0 (NA)	0	0 (NA)
**89**	0	0 (NA)	0	0 (NA)	0	0 (NA)

HPV type	Unvaccinated[2] N = 35		Vaccinated before age 15 years[3] N = 95		Vaccinated at age 15 years or later[4] N = 24	
	n[5]	PA % (95% CI)	n[5]	PA % (95% CI)	n[5]	PA % (95% CI)
**90**	0	0 (NA)	0	0 (NA)	0	0 (NA)
**91**	0	0 (NA)	0.01	0.02 (0-0.04)	0	0 (NA)

PA: proportional attribution, CI: confidence interval.

[1]Genotyping of cases reported during 2024 not complete at the time of data retrieval.

[2]Unvaccinated: Not registered with any HPV vaccine doses or the first dose received 365 days or less before they were first diagnosed with CIN2/3.

[3]Vaccinated before age 15 years: Cases who received at least one dose of HPV vaccine more than 365 days before they were first diagnosed with CIN2/3 and prior to the year they turned 15 years old.

[4]Vaccinated at age 15 years or later: Cases who received at least one dose of HPV vaccine 365 days or more before they were first diagnosed with CIN2/3, with the first dose administered the year they turned 15 years or later.

[5]Numbers are not whole due to weighting.
